# Cell-type-specific expression of tRNAs in the brain regulates cellular homeostasis

**DOI:** 10.1016/j.neuron.2024.01.028

**Published:** 2024-02-19

**Authors:** Mridu Kapur, Michael J. Molumby, Carlos Guzman, Sven Heinz, Susan L. Ackerman

**Affiliations:** 1 Department of Cellular and Molecular Medicine, University of California, San Diego, School of Medicine, La Jolla, CA 92093, USA; 2 The Howard Hughes Medical Institute; 3 Department of Medicine, Division of Endocrinology, School of Medicine, University of California, San Diego, La Jolla, CA 92093, USA; 4 Department of Bioengineering, Bioinformatics & Systems Biology Graduate Program, University of California, San Diego, La Jolla, CA 92093, USA; 5 Department of Neurobiology, Division of Biological Sciences, University of California, San Diego, La Jolla, CA 92093, USA; 6 These authors contributed equally; 7 Lead contact

## Abstract

Defects in tRNA biogenesis are associated with multiple neurological disorders, yet our understanding of these diseases has been hampered by an inability to determine tRNA expression in individual cell types within a complex tissue. Here, we developed a mouse model in which RNA polymerase III is conditionally epitope tagged in a Cre-dependent manner, allowing us to accurately profile tRNA expression in any cell type *in vivo*. We investigated tRNA expression in diverse nervous system cell types, revealing dramatic heterogeneity in the expression of tRNA genes between populations. We found that while maintenance of levels of tRNA isoacceptor families is critical for cellular homeostasis, neurons are differentially vulnerable to insults to distinct tRNA isoacceptor families. Cell-type-specific translatome analysis suggests that the balance between tRNA availability and codon demand may underlie such differential resilience. Our work provides a platform for investigating the complexities of mRNA translation and tRNA biology in the brain.

## In brief

Kapur et al. develop a conditional epitope-tagged Pol III mouse, allowing them to profile tRNA expression in individual nervous system cell populations *in vivo*. Their results highlight the heterogeneity of tRNA gene expression across the nervous system and provide a platform to investigate tRNA biology in the brain.

## Graphical Abstract

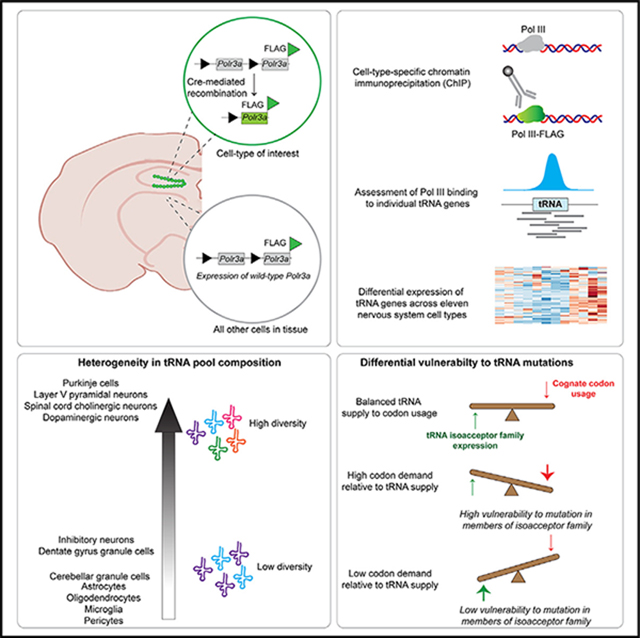

## INTRODUCTION

Transfer RNAs (tRNAs) play a critical role in mRNA translation, linking codons on mRNAs to their corresponding amino acids in the ribosome. Mammals have hundreds of highly conserved nuclear-encoded tRNA genes, organized into approximately 45 tRNA isoacceptor families based on their anticodon. These tRNA families can contain upwards of 50 genes, some of which encode tRNAs with identical mature sequences, while others have sequence differences in the body of the tRNA (tRNA isodecoders; Figure S1A).^1,2^ tRNAs are transcribed by RNA polymerase III (Pol III), which also transcribes other small non-coding RNAs, including 5S rRNA and U6 snRNA of the splicesome. tRNAs contain two internal promoters (A and B boxes), which are highly conserved across tRNA genes. The A and B boxes are recognized by TFIIIC,, leading to recruitment of TFIIIB to the 5’ end of the tRNA gene. TFIIIB, in turn, recruits Pol III to the transcription start site.^3^ Despite the use of shared transcription machinery at near-identical promoters and the absence of known Pol III tissue-specific transcription factors (TFs), the expression of individual tRNA genes varies greatly between tissues and different cell lines.^4–12^ For instance, *tRNA-Arg-TCT-4–1* (*n-Tr20*), a member of the tRNA^Arg^(TCT) family, is specifically and highly expressed in neurons, with little to no expression in glia.^7,12^ Loss of this tRNA alters seizure susceptibility and synaptic transmission in mice, highlighting its essential role in neuronal function.^7^

Disruption of tRNA biogenesis and metabolism has devastating consequences, resulting in diverse neurological and neurodegenerative diseases.^13–16^ Despite this, our understanding of tRNA expression and regulation in the brain remains poor. tRNAs are difficult to sequence and quantify because of their secondary structure and modifications. In addition, the similarity between tRNA genes is a computational challenge. Although there has been a recent surge in the development of RNA sequencing methods that overcome (with varying success) the major hurdles to tRNA quantitation, these studies have largely focused on cell lines or bulk tissue.^6,17–21^ The relatively weak correlation between tRNA abundances determined by the different methodologies^6^ and the rapidly evolving library preparation protocols make it difficult to select a “benchmark” method. In addition, current methodologies cannot uniquely map identical tRNA genes despite evidence that identical tRNA genes can differ in both spatial and temporal expression.^11^ Some of these issues are circumvented by chromatin immunoprecipitation sequencing (ChIP-seq) of Pol III machinery, which quantifies Pol III occupancy on tRNA genes and is not biased by tRNA modifications or structure. ChIP-seq reads often include flanking genomic sequence, and thus, tRNA genes with identical mature sequences can be successfully resolved.^5,8,22,23^ However, all these approaches are limited by the fact that complex tissues, including the brain, are composed of multiple cell types that may have unique tRNA expression profiles.

To address these issues, we developed a conditional Pol III knockin (KI) mouse line that allows for investigation of *in vivo* cell-type-specific tRNA expression using ChIP-seq. Conditional activation of this allele by an appropriate Cre recombinase leads to the expression of epitope-tagged Pol III that can be immunoprecipitated specifically from the target cell population. Our model allows for the successful determination of expression patterns of tRNA genes with identical mature sequences, even in rare cell types. We investigated tRNA expression in seven neuronal and four non-neuronal cell types in the mouse nervous system to create a cell-type-specific tRNA atlas. We show that while the expression of individual tRNA genes varies widely between all analyzed cell types, there are core sets of tRNA genes enriched in either neuronal or non-neuronal cells whose expression serves as a signature of these cell classes. We find that the tRNA pool in certain neuronal populations is more diverse than that of other neuronal and non-neuronal cells. Finally, we assessed the impact of tRNA mutations *in vivo* using a sensitized mouse background lacking the translational GTPase *Gtpbp2*^12^ and discovered that an interplay of tRNA expression level and cognate codon usage determines the vulnerability of neuron populations to tRNA mutations.

## RESULTS

### Epitope-tagged POLR3A can be used to accurately profile tRNA expression

**Figure 1.**
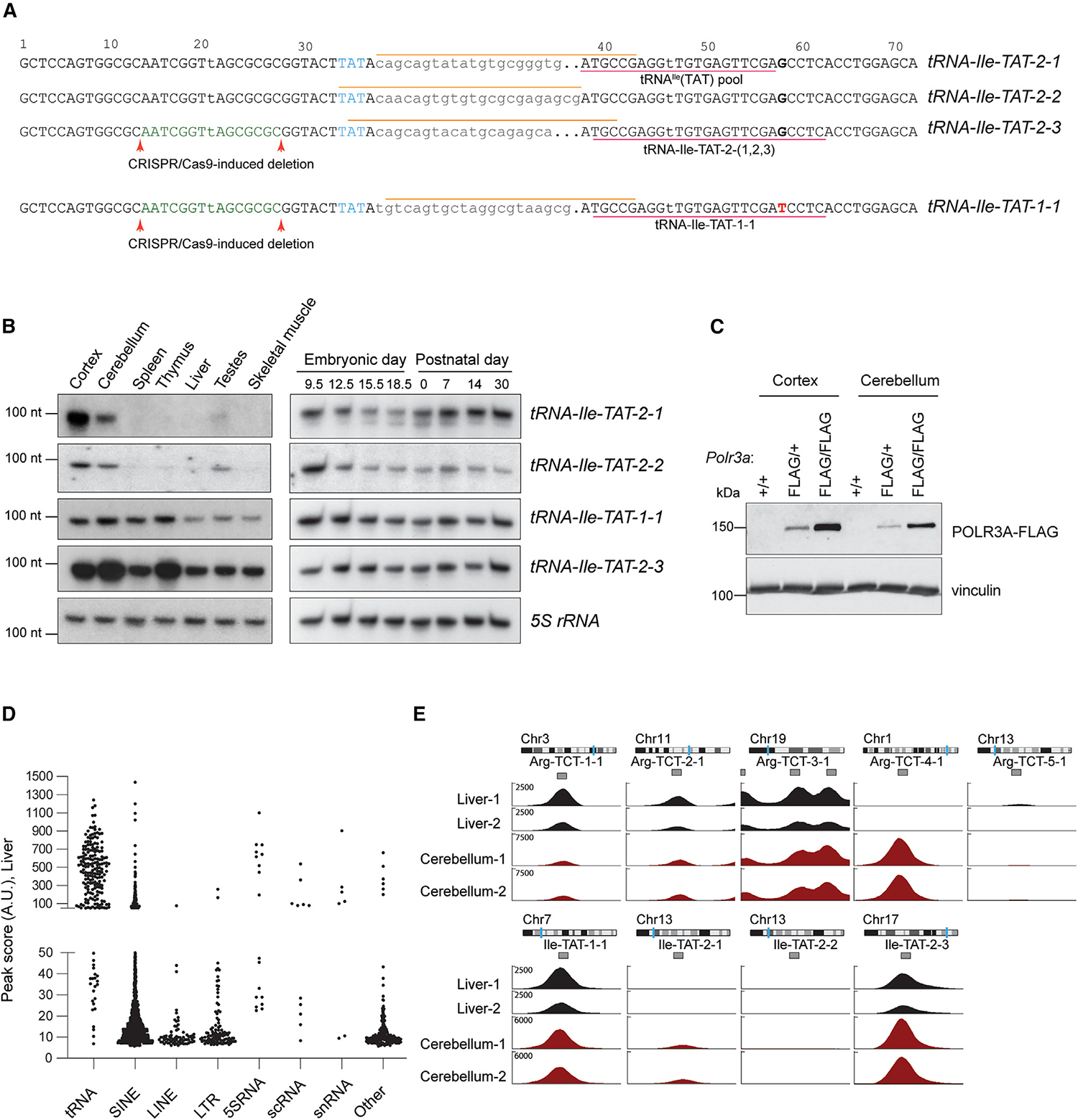
Epitope-tagged POLR3A can accurately profile tRNA expression *in vivo* (A) Sequences of the mouse tRNA^Ile^(TAT) genes with the anticodon (blue), introns (lowercase), and the SNP in *tRNA-Ile-TAT-1–1* (red) highlighted. The northern probes for pre-tRNAs and for mature tRNA sequences are indicated by orange and pink lines, respectively. (B) Northern blots of tRNA^Ile^(TAT) isodecoders using pre-tRNA probes in adult mouse tissues (left) and developing mouse brain (E9.5: whole head) (right). Loading control: 5S rRNA. Exposure time is optimized to detect signal from at least one tissue/age, and the relative expression of different genes cannot be compared. (C) Western blot of Sox2-Cre-mediated incorporation of the 3x-FLAG tag in POLR3A. Loading control: vinculin. (D) POLR3A-FLAG occupancy peak score distribution in mouse liver separated by gene annotation. Each point is a detected peak. (E) Representative ChIP-seq peaks for Pol III-bound loci at tRNA^Arg^(TCT) and tRNA^Ile^(TAT) gene families. Biological replicates are shown. See also Figure S1 and Table S1.

The issues that hinder the investigation of tRNA biology in the brain are highlighted by the tRNA^Ile^(TAT) family. In mice, this family consists of four high-confidence tRNA genes, three of which have identical mature sequences (*tRNA-Ile-TAT-2–1*, *-2–2*, and *-2–3*), while the fourth differs by only one nucleotide (*tRNA-Ile-TAT-1–1*) (Figure 1A). We found striking differences in the spatial and developmental expression of these tRNAs (Figure 1B). Northern blot analyses of the precursor tRNAs (using probes that overlap with the intron) in adult mice reveal that while *tRNA-Ile-TAT-1–1* and *tRNA-Ile-TAT-2–3* are widely expressed, *tRNA-Ile-TAT-2–1* and *tRNA-Ile-TAT-2–2* are mainly expressed in the brain, with weaker expression in the testes. In addition, the expression of the two brain-enriched tRNAs differs dramatically during development.

Strikingly, we have previously shown that *tRNA-Ile-TAT-2–3* composes approximately 90% of the pool of identical *Ile-TAT-2* tRNAs in the brain, indicating that these brain-specific tRNAs are only weakly expressed.^7^ However, rather than simply being lowly expressed, the expression of *tRNA-Ile-TAT-2–1* and *-2–2* may be restricted to specific neural cell populations and masked by bulk analysis of the brain. Investigation of this possibility requires analysis of gene- and cell-type-specific tRNA expression.

We chose the *Polr3a* gene, which encodes a DNA binding subunit of Pol III, as the target for conditional epitope tagging in mice using CRISPR-Cas9-mediated homology-directed repair (Figure S1B). In the targeted allele (*Polr3a-cKI*), we introduced *loxP* sites flanking the last exon (exon 31) of *Polr3a*, followed by an additional epitope-tagged (3x-FLAG) exon 31. Upon Cre-mediated recombination, the wild-type exon is excised, resulting in expression of the FLAG-tagged protein, whereas in the absence of Cre recombination, wild-type POLR3A protein is expressed.

To test the functionality of the *Polr3a*^FLAG^ allele, we crossed the *Pol3a-cKI* mice to an SRY-box-containing gene 2 (*Sox2*)-Cre recombinase mouse activating the *Polr3a*^FLAG^ allele in all tissues. *Polr3a*^FLAG/FLAG^ homozygous animals are viable, fertile, and indistinguishable from *Polr3a*^+/+^ littermates. Western blot analysis using an anti-FLAG antibody revealed POLR3A expression at the expected size, and signal intensity scaled with allele zygosity (Figure 1C).

To ensure that introduction of an epitope tag did not impact chromatin binding by POLR3A, we used an anti-FLAG antibody to perform ChIP-seq on the liver and cerebellum of POLR3A^FLAG/FLAG^ mice. The FLAG ChIP-seq signal was enriched at loci producing known Pol III-transcribed genes consistent with ChIP-seq datasets generated using antibodies against endogenous POLR3A (Figures 1D and S1C; Tables S1A and S1B).^5,8,22^ On average, the greatest number of peaks, though with low peak enrichment scores, mapped to short interspersed nuclear elements (SINEs), transposable elements that make up ~8% of the mouse genome,^24^ while peaks with the highest scores were located at tRNA genes. Analysis of the POLR3A^FLAG^ peaks recapitulated the tissue-specific expression of the neuron-specific gene *tRNA-Arg-TCT-4–1*, as a peak at this gene was detected in the cerebellum but not in the liver (Figure 1E; Tables S1A and S1B). The *tRNA-Arg-TCT-4–1* peak was higher than that detected on other members of the tRNA^Arg^(TCT) family in the cerebellum, consistent with it being the major member expressed in the brain. Finally, the peaks on *tRNA-Ile-TAT-1–1* and *-2–3* were much higher than those on *tRNA-Ile-TAT-2–1* and *-2–2*, in line with our previous finding that these genes are lowly expressed members of the tRNA^Ile^(TAT) family.

We next examined POLR3A^FLAG^ occupancy at individual tRNA genes. Approximately 60% of tRNA genes were occupied by Pol III (Table S1C). Only one of the 64 low-confidence tRNA genes was strongly occupied by Pol III, consistent with the prediction that the majority of these tRNAs are not functional in mRNA translation.^25^ The occupancy of tRNA genes from our liver dataset strongly correlated with previously published mouse liver POLR3A ChIP-seq datasets, further validating that the introduction of the epitope tag in POLR3A does not alter Pol III binding to tRNA genes and establishing the utility of the *Polr3a-cKI* mouse model for Pol III ChIP (Figure S1D).^5,8,22^

Finally, we compared our tRNA abundance measurements inferred by ChIP-seq with data obtained by tRNA sequencing (QuantM-tRNA-seq; Table S1D).^6^ We observed multiple tRNA genes that were detected as highly transcribed by Pol III ChIP-seq but had few reads in the QuantM-tRNA-seq dataset, likely resulting from inefficient amplification of heavily modified tRNAs or errors in accurately mapping reads. Despite these outliers, we found that tRNA abundance determined by POLR3A^FLAG^ occupancy in the liver and cerebellum was indeed positively correlated with levels determined by QuantM-tRNA-seq (Figure S1E).

### Creation of a nervous system tRNA atlas using cell-type-specific Pol III ChIP-seq

We next investigated tRNA expression in 11 cell types (seven neuronal and four non-neuronal) that represent a small portion of the cellular diversity of the nervous system. *Polr3a*^cKI^ mice were crossed to appropriate Cre recombinase lines to allow for specific isolation of POLR3A^FLAG^/chromatin complexes from the cell type of interest (Table S2). 289 tRNA genes were occupied by Pol III in at least one of the 11 cell types (Tables S3A–S3C). The correlation of tRNA expression between biological replicates was consistently high, with an average Pearson correlation coefficient of 0.92 (Table S3C). Approximately 90% of the 176 tRNA genes that were poorly expressed in the adult nervous system appear transcriptionally inactive across different developmental stages and cell types, including stem cells and cancer cells (Table S3D).^5,23,26–28^ The majority of these inactive tRNAs (>65%) are classified as high-confidence tRNA genes, suggesting that they could potentially be transcribed in physiological conditions not examined here. Alternatively, growing evidence suggests that tRNAs may have extra-transcriptional functions, acting as chromatin insulators.^29–33^

**Figure 2.**
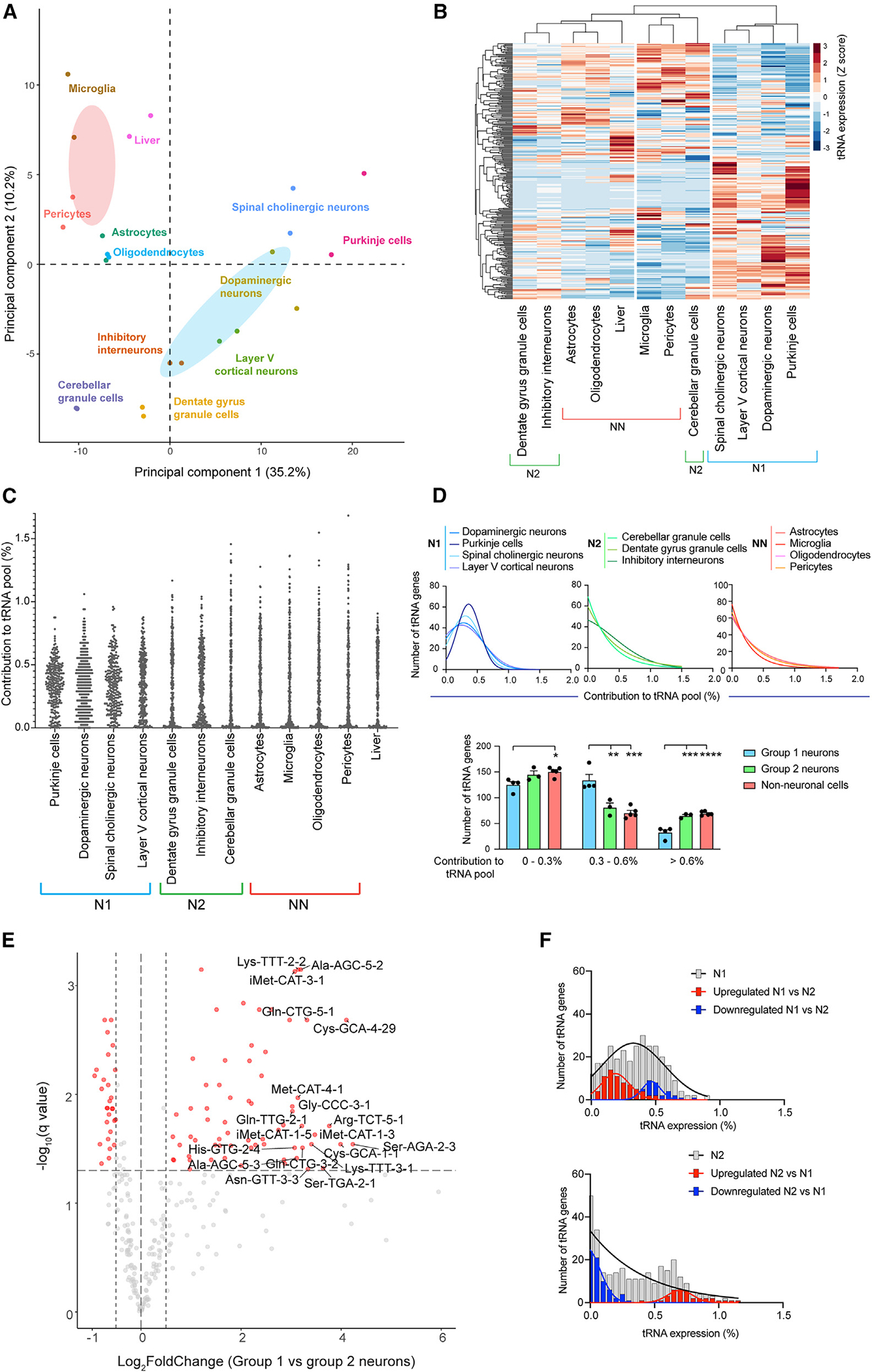
A comprehensive atlas reveals remarkable variation in tRNA expression across distinct cell types in the nervous system (A) Principal-component analysis (PCA) of tRNA expression in nervous system cell types and liver. Two biological replicates for each cell type and 95% confidence ellipses for neuronal (blue) and non-neuronal (pink) cells are shown. (B) Heatmap of the *Z* scores of tRNA gene expression. Columns are clustered based on Euclidean distance. N1, group 1 neurons; N2, group 2 neurons; NN, non-neuronal cells. (C) Scatterplot representing the relative contribution of each tRNA gene to the global tRNA pool. (D) Frequency distribution of relative contributions of all expressed tRNA genes to the global tRNA pool in group 1 neurons (N1), group 2 neurons (N2), and non-neuronal cells (NN). One-way ANOVA with Tukey post-test. *p s≤ 0.05, **p ≤ 0.01, ***p ≤ 0.001, ****p ≤ 0.0001, mean + SEM. (E) Differentially expressed tRNA genes between group 1 and group 2 neurons. tRNA genes with significantly altered expression are highlighted in red. Genes with |log_2_FoldChange| > 3 are labeled with the gene name. Horizontal line: q value = 0.05, vertical lines: log_2_FoldChange = 0.5 and −0.5. (F) Frequency distribution of the relative contributions of tRNA genes to the global tRNA pool. All tRNAs (gray), upregulated (red), downregulated (blue). See also Figure S2 and Tables S2 and S3.

The contribution of individual tRNA genes to the global tRNA pool varied greatly between the examined nervous system cell types (Tables S3A and S3B). Principal-component analysis (PCA) of tRNA expression revealed distinct separation of the cell populations, with neuronal and non-neuronal cells forming non-overlapping clusters (Figure 2A). Interestingly, among the seven examined neuron populations, Purkinje cells, dopaminergic neurons, layer V cortical neurons, and spinal cord cholinergic neurons (group 1 neurons) clustered together, indicative of similarities in their tRNA expression (Figure 2B). Closer examination revealed that the expression of tRNA genes in group 1 neurons was shifted toward the median relative to their expression in other neuron (group 2: cerebellar and dentate gyrus granule cells and inhibitory interneurons) and non-neuronal cell populations—that is, the tRNA pool in group 1 neurons consists of more moderately expressed tRNA genes (Figures 2C and 2D). Differential analysis of tRNA expression between group 1 and group 2 neurons identified 95 tRNA genes with significantly altered expression between these groups (q value ≤ 0.05 and |log_2_FoldChange| ≥ 0.5), with pronounced skewing toward upregulation in group 1 relative to group 2 neurons (Figure 2E). Strikingly, despite significant increases in their expression in group 1 relative to group 2 neurons, the majority of the upregulated tRNA genes were still relatively low contributors to the group 1 tRNA pool (Figure 2F). In contrast, tRNA genes whose expression is upregulated in group 2 relative to group 1 neurons were among the top contributors to the group 2 tRNA pool. Together, this is consistent with a shift toward a more diverse tRNA gene pool in group 1 neurons.

We next investigated how these changes in tRNA expression impacted the expression of individual tRNA isoacceptor families. We observed that the percentage of tRNA genes contributing significantly to anticodon pools (>5% of the isoacceptor family) in group 1 neurons was significantly higher than that in group 2 neurons or non-neuronal cells. This effect was observed across the size range of tRNA families, indicating a global increase in the heterogeneity of tRNA isoacceptor pools in group 1 neurons (Figures S2A and S2B). Increased expression of minor members of a tRNA family could alter the diversity of the tRNA isoacceptor family, with or without changing the total levels of the family. We found that 15 tRNA families were differentially expressed between group 1 and group 2 neurons; however, none of them met our minimum fold-change threshold (|log_2_FoldChange| ≥ 0.5), indicating that the changes in tRNA gene expression serve largely to alter the composition of tRNA isoacceptor pools rather than changing their levels (Figure S2C).

### Cerebellar granule cells express a distinct tRNA repertoire

Although the analyzed neuron populations can be categorized into groups based on their shared features, each individual neuron population still appears to express a unique tRNA repertoire (Figures 2A and 2B). To identify tRNA genes that drive the separation of individual neuronal types, we first examined genes with the highest variance in expression across all seven neuron populations (Figures S3A and S3B). Most of the top 25 high-variance tRNA genes (18/25) were also identified as differentially expressed between group 1 and group 2 neurons (Figure 2E), and cerebellar granule cells were the primary drivers of variance in expression (Figure S3A). Consistent with this, cerebellar granule cells had the largest Euclidean distance from all other neuronal populations, including the other two group 2 neuron populations (Figure S3C). In contrast, the variation in tRNA expression across group 1 neurons was relatively small (Figure S3D; see also Figures S2B and S3B), as apparent by the similar Euclidean distance across these cell types (Figure S3C).

Finally, we examined tRNAs with the highest variance in expression across the non-neuronal populations (Figure S3E). Expression of these tRNAs was similar in astrocytes and oligodendrocytes, consistent with their shared developmental origin. Interestingly, only three high-variance tRNAs (*tRNA-Glu-TTC-2–1*, *tRNA-Leu-TAG-3*-1, and *tRNA-Ala-TGC-4–1*) were also identified as high variance in neuronal populations, indicating that transcriptional variance may be constrained within certain cell populations and is unlikely to be noise.

### Identification of neuronal and non-neuronal tRNA gene signatures

**Figure 3.**
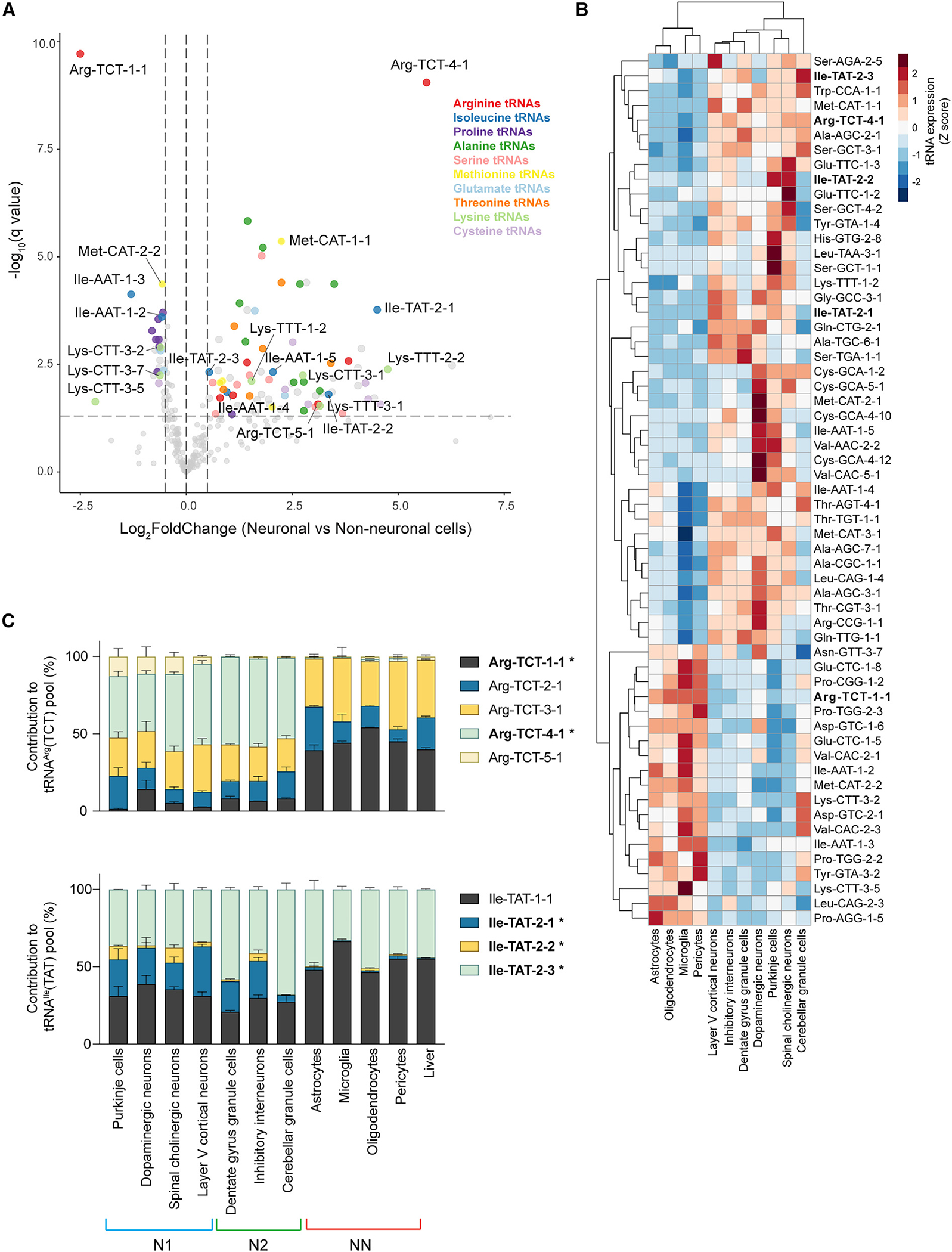
The tRNA repertoire in neuronal cells differs from that expressed in non-neuronal cells (A) Differentially expressed tRNA genes between neuronal and non-neuronal cells. Select tRNA genes with significantly altered expression are highlighted in the indicated colors. Horizontal line: q value = 0.05, vertical lines: log_2_FoldChange = 0.5 and −0.5. (B) Heatmap of expression *Z* scores of tRNAs that are differentially expressed (q value ≤ 0.05 and |log_2_FoldChange| ≥ 0.5) between neuronal and non-neuronal cells, but not between group 1 and group 2 neurons. Columns are clustered based on Euclidean distance. tRNA^Arg^(TCT) and tRNA^Ile^(TAT) genes are labeled in bold. (C) Expression of tRNA^Arg^(TCT) and tRNA^Ile^(TAT) genes (mean + SEM). Differentially expressed genes between neurons and non-neuronal cells are bolded and labeled with an asterisk. See also Figures S3 and S4 and Table S3.

Despite the differences in tRNA expression between individual cell types in the nervous system, the distinct clustering of neuronal and non-neuronal cells (Figure 2A) suggests the existence of a core set of tRNAs that are differentially expressed between these cell categories. We therefore performed differential analysis of tRNA expression between the seven neuronal and four non-neuronal cell types, which identified 109 differentially expressed tRNA genes (|log_2_FoldChange| ≥ 0.5; Figure 3A). Approximately half of these genes were previously identified as differentially expressed between group 1 and group 2 neurons (Figure 2E). Indeed, expression of these tRNAs in group 2 neurons was similar to that in non-neuronal cells, indicating that their differential expression between neuronal and non-neuronal cells is specifically driven by group 1 neurons (Figure S4A). Thus, we focused on the remaining 59 differentially expressed tRNA genes to identify pan-neuronal patterns in tRNA expression (Figure 3B).

As expected,^7,12^ *tRNA-Arg-TCT-4–1* was one of the most significantly upregulated tRNAs in neurons relative to non-neuronal cells (Figures 3A–3C). Another isodecoder, *tRNA-Arg-TCT-5–1*, was also upregulated in group 1 neurons relative to non-neuronal cells (Figures 3C and S4A). Interestingly, *tRNA-Arg-TCT-1–1* is significantly upregulated in non-neuronal cells, where it constitutes >50% of the tRNA^Arg^(TCT) pool (Figures 3A–3C). While other isoacceptor families (including Ile-AAT, Met-CAT, and Ala-TGC) also have members that show significant but directionally opposed changes in expression between neurons and non-neuronal cells, the differentially expressed tRNAs either are minor contributors to large tRNA families or show lower fold changes in expression (Figures S3B and S4B). Thus, the tRNA^Arg^(TCT) family is the only isoacceptor family with a complete switch in its major isodecoder between neuronal and non-neuronal cells.

Consistent with their brain-specific expression (Figure 1B), *tRNA-Ile-TAT-2–1* and *-2–2* were also identified as neuron-specific genes (Figures 3A–3C). However, in contrast to *tRNA-Arg-TCT-4–1*, which is a major contributor to the tRNA^Arg^(TCT) pool in all analyzed neuronal populations, *tRNA-Ile-TAT-2–1* is barely expressed in cerebellar granule cells and makes up only a quarter of the tRNA^Ile^(TAT) pool in other neuronal populations (Figure 3C). *tRNA-Ile-TAT-2–2* is a minor contributor to the tRNA^Ile^(TAT) pool, likely only expressed in two of the neuronal populations: namely, Purkinje cells and spinal cord cholinergic neurons.

We also observed coordinated up- or downregulation of multiple tRNA genes belonging to the same tRNA isotype (Figures 3A and 3B). For instance, alanine tRNA genes belonging to the tRNA^Ala^(AGC), tRNA^Ala^(CGC), and tRNA^Ala^(TGC) family were significantly upregulated in neuronal relative to non-neuronal cells (Figures 3A, S2B, and S3B). In addition, tRNA genes from multiple serine and threonine isoacceptor families were also enriched in neurons. In contrast, non-neuronal cells showed increased expression of six tRNAs from the three proline tRNA isoacceptor families relative to most neuronal cells.

Finally, we identified multiple tRNA genes that were differentially expressed between neuronal and non-neuronal cells but whose expression in the liver did not align either with glial cells or pericytes. For example, *tRNA-Met-CAT-1–1* has higher expression in neurons and liver relative to non-neuronal cells in the nervous system (Figures S4B and S4C). These tRNAs would likely be overlooked in differential expression analyses on the tissue level and were only identified because of the cell-type resolution of our approach.

In summary, despite the diversity of tRNA expression between individual cell populations, we have identified tRNA genes whose expression level serves as general markers for neuronal or non-neuronal cell-type identity in the nervous system.

### Differential modulation of tRNA isoacceptor and isotype families in neuronal and non-neuronal cells

**Figure 4.**
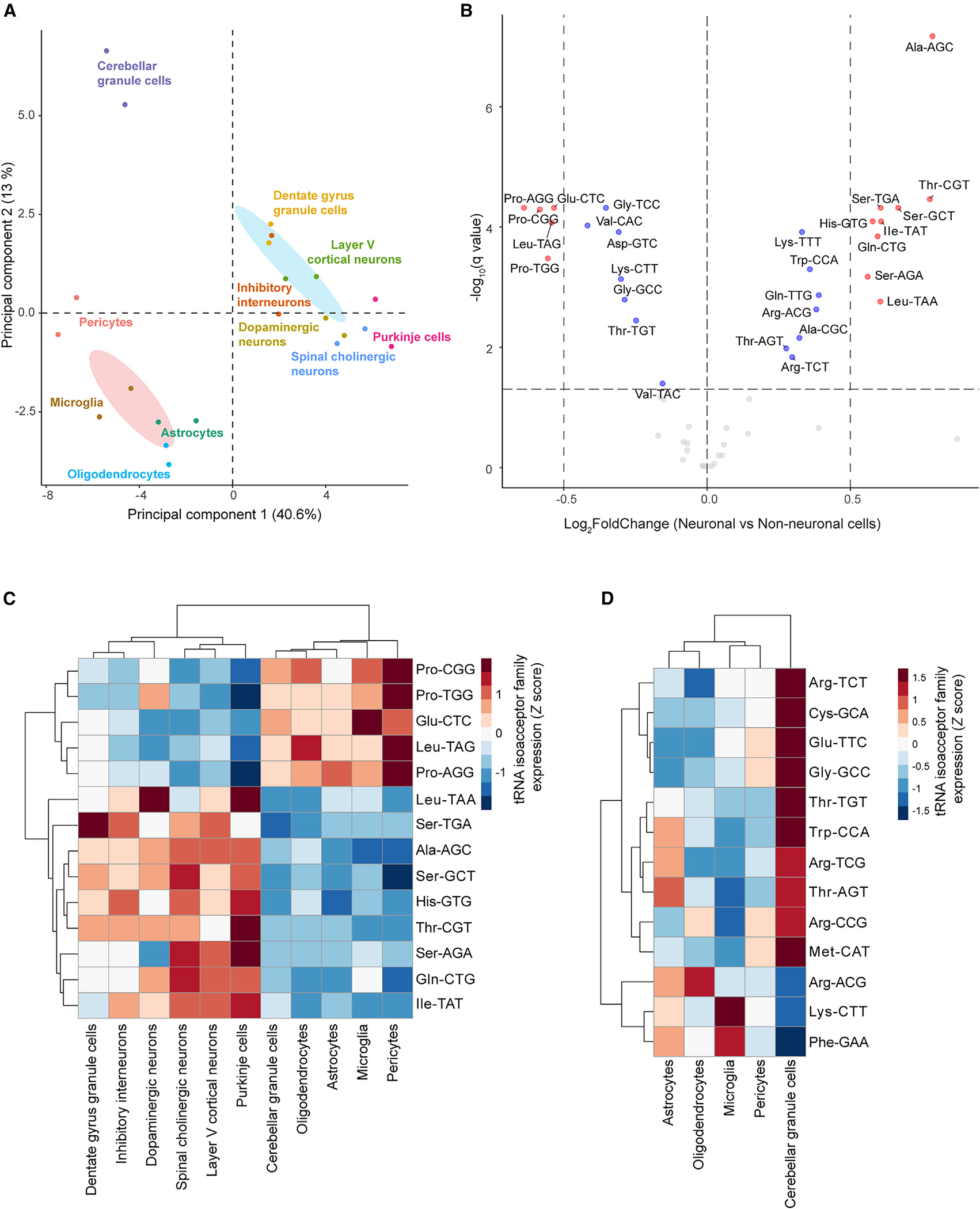
Differential expression of tRNA isoacceptor and isotype families in neuronal and non-neuronal cells in the nervous system (A) Principal-component analysis (PCA) of tRNA isoacceptor expression. Two biological replicates for each cell type and 95% confidence ellipses for neuronal (blue) and non-neuronal (pink) cells are shown. (B) Differentially expressed tRNA isoacceptor families between neuronal (except cerebellar granule cells) and non-neuronal cells in the nervous system. Horizontal dashed line: q value = 0.05. Vertical dashed lines: log_2_FoldChange = 0.5 and −0.5. tRNA families with |log_2_FoldChange| > 0.5 are red, while tRNA families with |log_2_FoldChange| < 0.5 are blue. (C) Heatmap of *Z* scores for expression of tRNA isoacceptor families that are differentially expressed (q value ≤ 0.05 and |log_2_FoldChange| > 0.5) between neuronal and non-neuronal cells. (D) Heatmap of the *Z* scores for expression of tRNA isoacceptor families for which cerebellar granule cell expression deviates from that of non-neuronal cells. See also Figure S5.

The expression of tRNA isoacceptor and isotype families has been reported to be more stable across tissues and developmental stages compared to the expression of individual tRNA genes.^5,8^ Indeed, despite differences in the expression of tRNA isodecoders between group 1 and group 2 neurons (Figure 2E), there were minimal changes in the expression of isoacceptor families (Figure S2C). Supporting this, most of the neuron populations clustered closely on PCA of isoacceptor levels (Figure 4A). However, we observed separation of neuronal and non-neuronal cells into distinct and non-overlapping clusters. As previously observed on the level of tRNA gene expression, cerebellar granule cells clustered apart from both other neuronal populations and non-neuronal cells, suggesting that they had distinct tRNA isoacceptor family profiles. Therefore, they were excluded from the neuronal group, and we performed differential analysis of tRNA isoacceptor family expression between the remaining six neuronal and four non-neuronal cell types. We identified 28 differentially regulated tRNA isoacceptor families, 14 of which met our minimum fold-change threshold (|log_2_FoldChange| ≥ 0.5; Figures 4B and 4C). The expression of these tRNA families in cerebellar granule cells aligned with non-neuronal cells rather than with the other neuron populations (Figure 4C). As has been previously reported,^34^ the tRNA^Ala^(AGC) family was the most significantly upregulated family in neurons relative to non-neuronal cells (Figure S2B). Neurons were also significantly enriched for eight other families, including three tRNA^Ser^ isoacceptor families (Figures S3B and S5A) and the tRNA^Thr^(CGT) family (Figure S5A). In contrast, non-neuronal cells were enriched for five isoacceptor families, including all three tRNA^Pro^ families (Figures S3B and S5A). Strikingly, we also identified multiple families whose levels in cerebellar granule cells differed from those of non-neuronal cells, further highlighting that cerebellar granule cells deviate from both the shared neuronal and non-neuronal tRNA isoacceptor profile (Figures 4D and S5B).

Finally, we investigated the impact of the differential expression of tRNA genes on the relative levels of tRNA isotypes. Cerebellar granule cells continued to cluster apart from all other neurons (Figure S5C). Differential analysis of tRNA isotype levels between the remaining six neuronal populations and non-neuronal cells identified 12 differentially expressed tRNA isotype families (Figure S5D); however, the majority of these had relatively small changes in level and only three families, namely tRNA-Gln, tRNA-His, and tRNA-Pro, met the minimum fold-change cutoff (|log_2_FoldChange| ≥ 0.5).

Together, our data indicate that while the expression of tRNA isoacceptor families is much less variable between nervous system cell types than that of individual tRNA genes, there are still clear differences in the expression of tRNA isoacceptor families between neuronal and non-neuronal cells. Furthermore, we found that cerebellar granule cells, the most abundant neuron population in the brain, have a distinct tRNA repertoire in terms of the composition and levels of tRNA isoacceptor families.

### Differential isoacceptor family abundance correlates with cognate codon usage in cell-type-specific genes

The extent to which the composition of the tRNA pool is tuned to the codon usage of certain genes to regulate their efficient and selective translation remains controversial.^26,35–42^ To elucidate the relationship between tRNA expression and codon usage in the translatome of nervous system cells, we generated cell-type-specific translatome libraries using the RiboTag mouse model.^43^ We generated libraries from four neuronal (cerebellar granule cells, dentate gyrus granule cells, layer V cortical neurons, and inhibitory interneurons) and two glial (astrocytes and microglia) cell types and used a previously generated library from Purkinje cells.^44^ Analysis of the expression of well-established marker genes^45–49^ reveals that our translatome data aligns with the expected profile of each cell population (Figure S6A).

**Figure 5.**
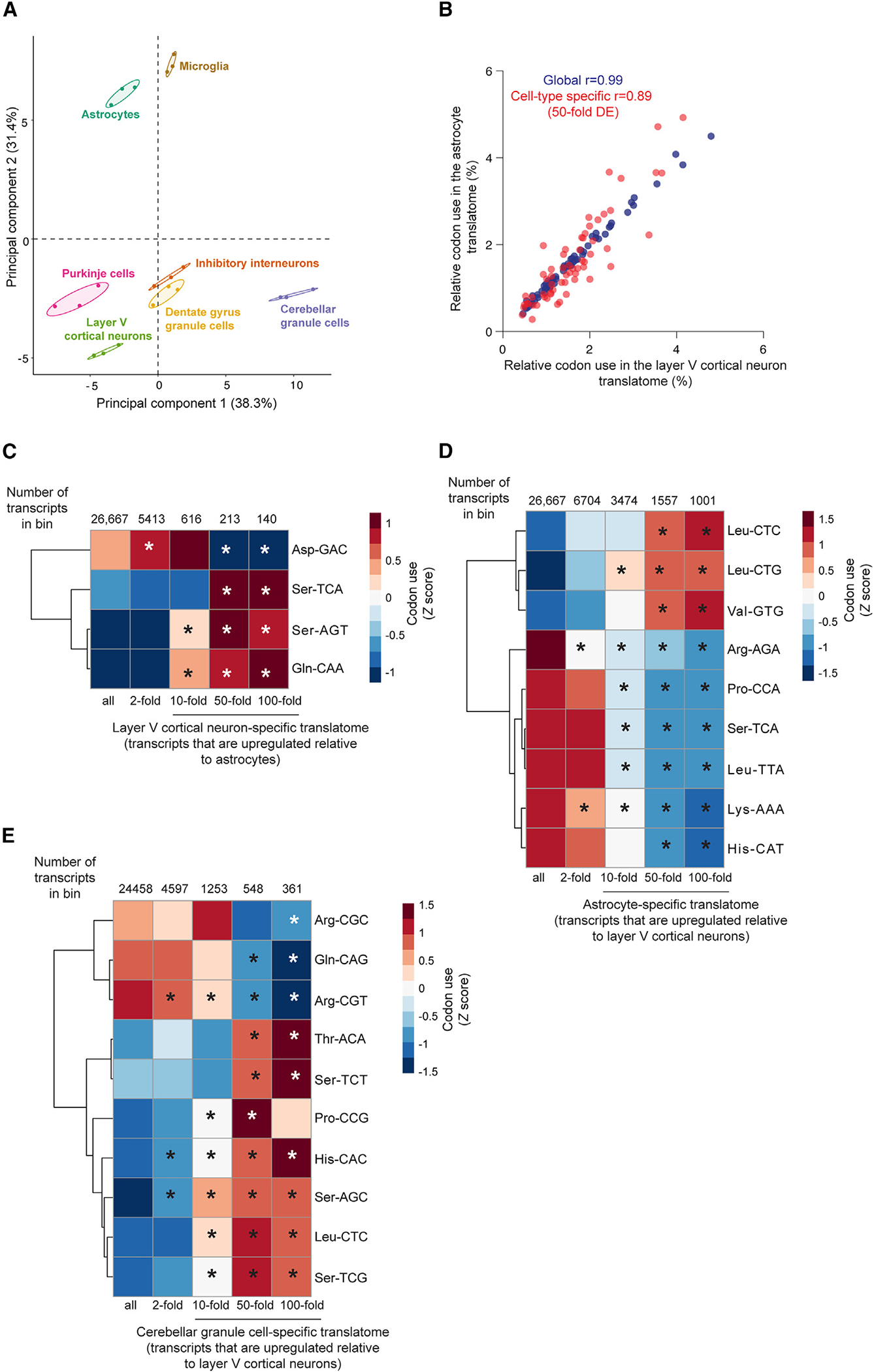
Differential isoacceptor family abundance partially correlates with cognate codon usage in cell-type-specific genes (A) Principal-component analysis (PCA) of translatome codon usage. Three biological replicates and 95% confidence ellipses are shown for each cell type. (B) Pearson correlation of codon use in the astrocyte and layer V cortical neuron translatome (blue) and in cell-type-specific genes that are differentially expressed at least 50-fold between the two cell types (red). (C–E) Heatmaps of *Z* scores for codon usage in the (C) cortical neuron-specific translatome (relative to astrocytes), (D) the astrocyte-specific translatome (relative to cortical neurons), and (E) the cerebellar granule cell-specific translatome (relative to cortical neurons). Bins in which the codon usage was significantly different than expected based on random sampling of the translatome are labeled with an asterisk. The number of transcripts in each bin is indicated. See also Figure S6 and Table S4.

We found that expression-weighted codon usage in the translatome (Table S4) was highly correlated between the different cell types (Pearson correlation coefficient > 0.99), consistent with previous studies that have suggested that codon usage in the transcriptome is extremely stable across different cell types and developmental stages.^5,34^ Despite the high degree of similarity in codon usage between the nervous system cell types, we did observe separation of the individual cell types in PCA—in particular, astrocytes and microglia separated from the different neuronal populations along principal component 2 (Figure 5A).

We then examined the correlation between the levels of tRNA isoacceptor families and codon usage in the translatome of the different cell types. To avoid making assumptions about non-canonical base-pairing efficiency, we omitted wobble pairs from our analysis and calculated the codon-anticodon correlation for each cell type (Figure S6B). Consistent with the fact that codon usage in the global translatome is extremely similar across the different cell populations, the correlation between tRNA isoacceptor levels and codon usage within each cell type was not significantly different than isoacceptor expression and the codon usage in other cell types (Mann-Whitney test of matched and mismatched Spearman rank correlation ρ, p value = 0.87), suggesting that the mRNA translatome of one cell type is translated with similar efficiency by the tRNA transcriptome of other cell types. However, the tRNA pools of certain cell types appear better tuned to the cellular translatome; for example, the tRNA pool in microglia correlates more strongly with global codon usage compared to that of cerebellar granule cells (Figure S6B).

Next, we investigated whether codon usage in cell-type-specific genes was coupled to differential tRNA isoacceptor family abundance between cell types. We first examined whether codon usage in cell-type-specific transcripts differed more than total translatomes between cell types. We found that although codon usage in the astrocyte and cortical neuron-specific translatomes (>50-fold upregulated in one cell relative to the other) were still strongly correlated to each other, the Pearson correlation coefficient was significantly lower than that for codon usage in global translatomes (Figure 5B, permutation test empirical p value ≤ 0.05). We next assessed which codons were over- or underrepresented in cell-type-specific genes relative to their usage in the global translatome. We identified three codons that were significantly overrepresented (empirical p value ≤ 0.05) in cortical neuron-specific transcripts (>50-fold upregulation relative to astrocytes): the Gln-CAA codon and the Ser-TCA and -AGT codons (Figure 5C). Strikingly, the cognate tRNA families for these codons (Gln-TTG, Ser-TGA, and Ser-GCT) are significantly enriched in layer V cortical neurons relative to astrocytes (Figures 4B and 4C). Conversely, Asp-GAC was the only significantly underrepresented codon in cortical neuron-specific transcripts relative to the global translatome, and the cognate tRNA isoacceptor family, Asp-GTC, is downregulated in neurons relative to non-neuronal cells.

Similarly, astrocyte-specific transcripts showed significantly increased usage of cognate or near-cognate codons of the astrocyte-enriched tRNA families Val-CAC and Leu-TAG and decreased usage for codons decoded by the Leu-TAA, Ser-TGA, Lys-TTT, Arg-TCT, and His-GTG tRNA isoacceptor families, whose levels are lower in astrocytes relative to cortical neurons (Figure 5D). However, this simple correlation did not hold true for all codons, as astrocyte-specific transcripts also had significantly decreased usage of Pro-CCA despite increased levels of the cognate tRNA family in astrocytes relative to cortical neurons. The presence of a proline codon in a ribosome’s A- or P-site has been established to slow down translation,^50^ and thus, higher levels of tRNA^Pro^(TGG) and its reduced usage in astrocyte-specific transcripts may contribute to their efficient translation in these cells. Together, these data suggest that the differential abundance of a subset of tRNA isoacceptor families between neuronal and non-neuronal cells may indeed be tuned to the usage of their cognate codons in cell-type-specific genes. For others, the impact of altered abundance of the isoacceptor family may be observed in ribosome decoding speed or efficiency.^34^

tRNA expression in cerebellar granule cells deviates from that in other neuronal populations (Figures S3, 4A, 4C, and 4D). We found that the usage of codons cognate to tRNA isoacceptor families with higher levels in cerebellar granule cells was significantly enriched in cerebellar granule-cell-specific transcripts(Figures 5E and S6C). Conversely, the usage of codons cognate to tRNA families with lower levels in cerebellar granule cells was reduced in these transcripts. However, we also observed increased usage of serine codons, His-CAC, and Phe-TTC in cerebellar granule cell-specific transcripts, even though the levels of the cognate isoacceptor families were lower in cerebellar granule cells than in cortical neurons or astrocytes. This suggests that the tRNA pool in cerebellar granule cells may not be optimized for codon-driven translational efficiency of cell-type-specific genes.

### Multigene tRNA families fail to efficiently compensate for genetic mutations

Most tRNA isoacceptor families contain multiple genes that have been hypothesized to be functionally redundant and provide protection against tRNA loss.^51^ However, we previously discovered that C57BL/6J (B6J) mice have a mutation that significantly reduces the levels of *tRNA-Arg-TCT-4–1* and thus the tRNA^Arg^(TCT) pool in the brain.^12^ Strikingly, this mutation is not functionally compensated for by the other tRNA^Arg^(TCT) isodecoders, though transgenic overexpression of these genes can rescue phenotypes caused by *tRNA-Arg-TCT-4–1* loss.^7^

**Figure 6.**
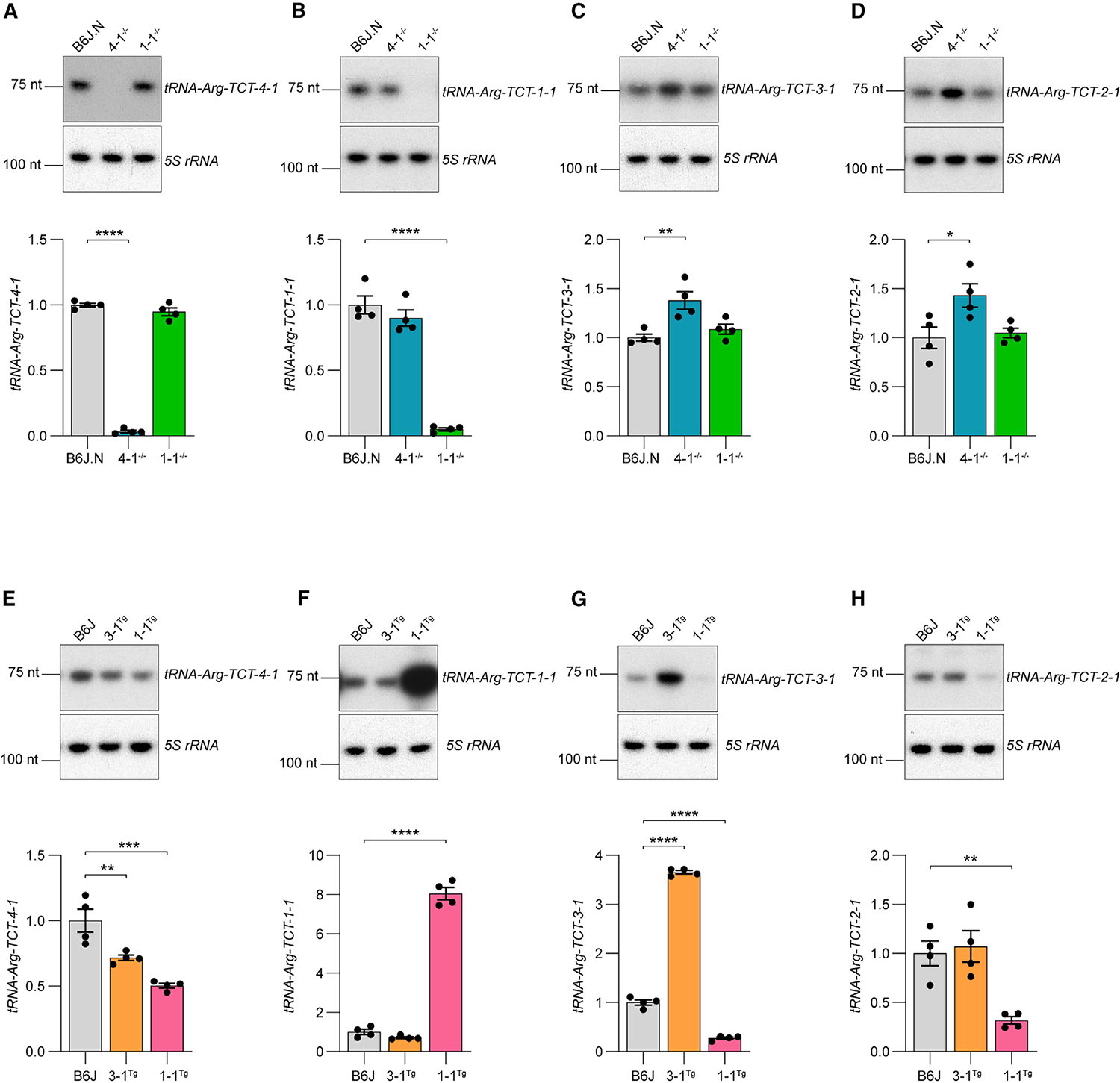
Multigene tRNA families fail to efficiently compensate for genetic mutations (A–D) Northern blots for expression of tRNA^Arg^(TCT) isodecoders in the cerebellum of *tRNA-Arg-TCT-4–1* and *tRNA-Arg-TCT-1–1* knockout mice compared to congenic B6J control mice in which the wild-type *tRNA-Arg-TCT-4–1* gene was transferred from B6N (B6J.N). (E–H) Northern blots for expression of tRNA^Arg^(TCT) isodecoders in the cortex of transgenic mice overexpressing either *tRNA-Arg-TCT-3–1* or *tRNA-Arg-TCT-1–1* compared to C57BL/6J (B6J) controls. (A-H) Bands were normalized to 5S rRNA (loading control) and quantified relative to respective controls. Mean ± SEM. One-way ANOVA with Tukey post-test *p ≤ 0.05, **p ≤ 0.01, ***p ≤ 0.001, ****p ≤ 0.0001. See also Figure S7.

To investigate the relationship between members of the tRNA^Arg^(TCT) family, we examined the expression of the other isodecoders in the cerebellum of *tRNA-Arg-TCT-4–1* knockout mice (Figures 6A–6D). We found that loss of *tRNA-Arg-TCT-4–1* caused a small but significant increase in the levels *of tRNA-Arg-TCT-3–1* and *tRNA-Arg-TCT-2–1* (Figures 6C and 6D). The level of *tRNA-Arg-TCT-1–1* was unchanged, indicating that this tRNA is relatively insensitive to loss of *tRNA-Arg-TCT-4–1* (Figure 6B). *tRNA-Arg-TCT-5–1* was not detectable by northern blot, consistent with its very low expression in cerebellar granule cells (Figure 3C). In addition, we found that loss of *tRNA-Arg-TCT-1–1*, which is not highly expressed in cerebellar granule cells (Figure 3C), did not impact expression of other tRNA^Arg^(TCT) isodecoders in the cerebellum, indicating that a substantial depletion of the tRNA isoacceptor family may be required to alter expression of other family members (Figures 6A–6D). Levels of other arginine tRNA isoacceptor families or of unrelated isoleucine tRNA families were not affected by loss of either *tRNA-Arg-TCT-4–1* or *tRNA-Arg-TCT-1–1* (Figures S7A–S7E), indicating that the upregulation of tRNA^Arg^(TCT) isodecoders represents a specific intra-family response rather than a global dysregulation of tRNA expression. However, this upregulation of tRNAs is inefficient and fails to restore basal levels of the tRNA^Arg^(TCT) pool.^12^

We next examined expression of tRNA^Arg^(TCT) isodecoders in B6J mice overexpressing either *tRNA-Arg-TCT-1–1* (~8-fold) or *tRNA-Arg-TCT-3–1* (~4-fold, Figures 6E–6H). Transgenic overexpression of these tRNAs significantly increased the tRNA^Arg^(TCT) pool in the brain relative to B6J mice, restoring it to the level seen in mice expressing wild-type *tRNA-Arg-TCT-4–1*.^7^ Overexpression of either *tRNA-Arg-TCT-1–1* or *tRNA-Arg-TCT-3–1* significantly reduced *tRNA-Arg-TCT-4–1* levels in the cortex relative to B6J mice (Figure 6E). In addition, overexpression of *tRNA-Arg-TCT-1–1* also significantly reduced levels of *tRNA-Arg-TCT-3–1* and *tRNA-Arg-TCT-2–1* (Figures 6G and 6H). Strikingly, overexpression of *tRNA-Arg-TCT-1–1* also significantly decreased levels of two other arginine tRNA isoacceptor families, but not unrelated isoleucine tRNA families (Figures S7F–S7J). Thus, tRNA overexpression results in more widespread changes in tRNA expression relative to that caused by *tRNA-Arg-TCT-4–1* loss.

### The interplay between tRNA expression and codon usage may contribute to vulnerability to tRNA mutations

We have previously shown that mutation of *tRNA-Arg-TCT-4–1* causes ribosome stalling, alters translation signaling pathways, and disrupts synaptic physiology.^7,12^ Our work indicates that these phenotypes stem from depletion of the tRNA^Arg^(TCT) pool rather than from loss of a specific function of *tRNA-Arg-TCT-4–1*, suggesting that the mammalian nervous system may be vulnerable to depletion of other tRNA isoacceptor families.

**Figure 7.**
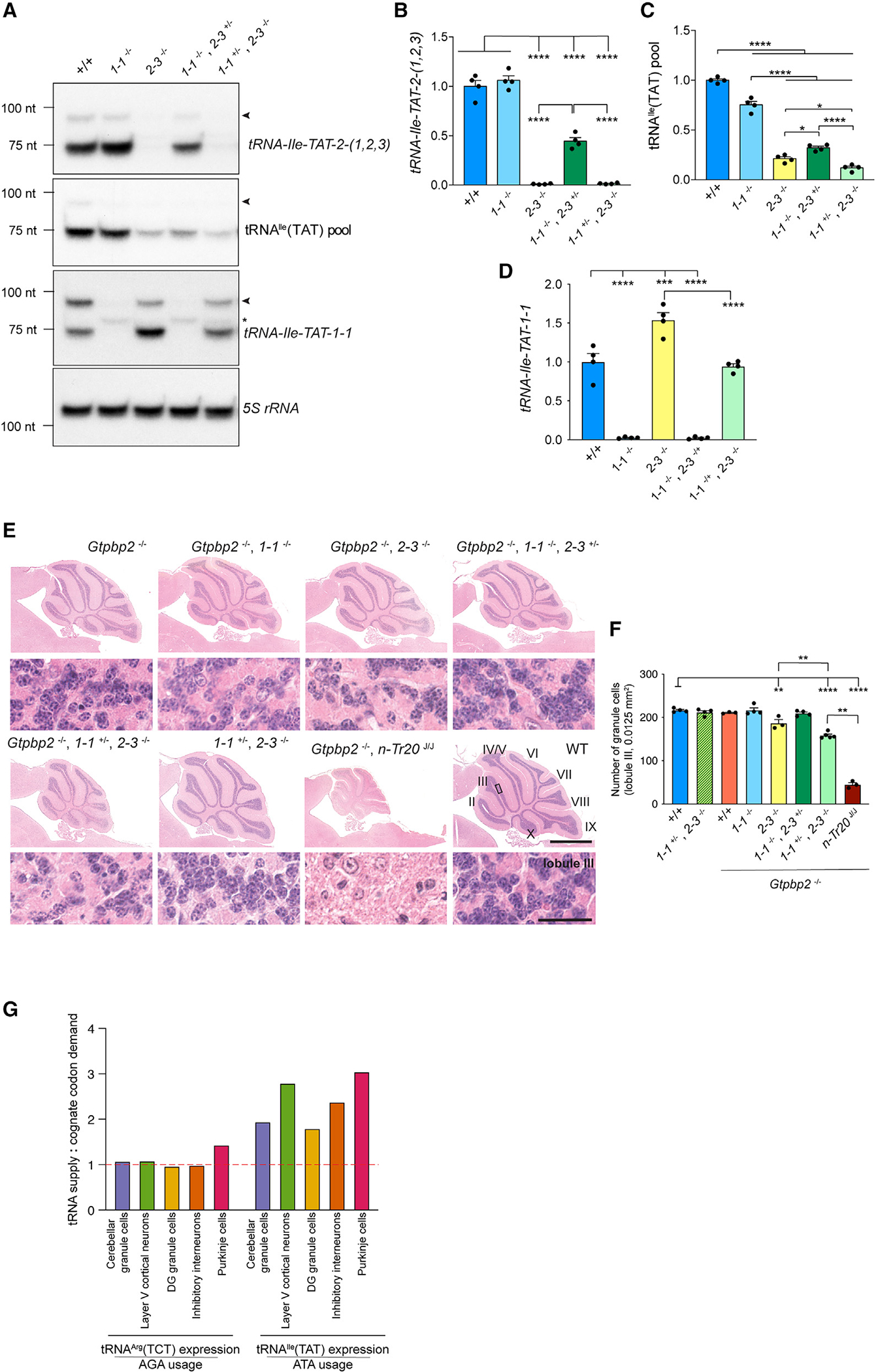
Differences in codon use may underlie the resilience of cerebellar granule cells to depletion of the tRNA^Ile^(TAT) family relative to the tRNA^Arg^(TCT) family (A) Northern blots of the expression of tRNA^Ile^(TAT) genes and the tRNA^Ile^(TAT) pool in the cerebellum of B6J.N mice (wild-type, +/+), *tRNA-Ile-TAT-1–1*^−/−^ mice (1–1^−/−^), *tRNA-Ile-TAT-2–3*^−/−^ mice (2–3^−/−^), and compound mutants homozygous for loss of one of these tRNAs and heterozygous for another (1–1^−/−^, 2–3^+/−^ and 1–1^+/−^, 2–3^−/−^). Loading control: 5S rRNA. Black arrowheads indicate the precursor or immature tRNA bands, and an asterisk marks the truncated transcript produced from the mutant *tRNA-Ile-TAT-1–1* locus. (B–D) tRNA levels are quantified relative to wild-type (B6J.N) mice. Data are represented as mean + SEM. (E and F) Depletion of the tRNA^Ile^(TAT) pool in the context of *Gtpbp2* loss causes cerebellar granule cell degeneration. (E) Hematoxylin and eosin-stained (H&E) sagittal sections of cerebella from 12-week-old mice except *Gtpbp2*^−/−^; *tRNA-Arg-TCT-4–1*^B6J/B6J^ (*Gtpbp2*^−/−^, *n-Tr20* ^J/J^), which is shown at 8 weeks. Lower panels are higher-magnification images of lobule III (black rectangle in wild-type). Scale bars, 1 mm (top panel), 50 μm (bottom panel). Cerebellar lobules are indicated by Roman numerals. (F) Quantification (mean + SEM) of cerebellar granule cells in lobule III of the indicated genotypes. (G) Ratio of the relative tRNA isoacceptor family levels to the expression-weighted usage of the cognate codon in the translatome. (B–D and F) One-way ANOVA with Tukey post-test. *p ≤ 0.05, **p ≤ 0.01, ***p ≤ 0.001, ****p ≤ 0.0001. See also Figure S8.

To investigate this hypothesis, as well as validate our measurements of tRNA expression generated using the *Polr3a*^cKI^ mice, we targeted the four-member tRNA^Ile^(TAT) family, composed of *tRNA-Ile-TAT-2–1*, *-2–2*, and *-2–3* and *tRNA-Ile-TAT-1–1* via CRISPR-Cas9-mediated mutagenesis. We focused on the cerebellum, which has a lower glial content than other regions of the brain and consists largely of cerebellar granule cells (>99% of the neurons in the cerebellum).^52^ Our Pol III ChIP-seq data (Figure 3C; Table S3) estimates that the tRNA^Ile^ (TAT) pool in cerebellar granule cells consists of *tRNA-Ile-TAT-2–3* (67%) and *tRNA-Ile-TAT-1–1* (29%), with minimal expression of the other two genes (*tRNA-Ile-TAT-2–1* and *-2–2*). Northern blot analyses of *tRNA-Ile-TAT-2–3* mutant mice using a probe that recognizes all three identical *tRNA-Ile-TAT-2-(1,2,3)* genes (Figure 1A), but not *tRNA-Ile-TAT-1–1*, gave no signal, demonstrating that *tRNA-Ile-TAT-2–1* and *-2–2* are indeed lowly expressed (Figures 7A and 7B). Loss of *tRNA-Ile-TAT-2–3* reduced the tRNA^Ile^(TAT) pool to approximately 21% of that in wild-type mice, reflecting expression of *tRNA-Ile-TAT-1–1*, the levels of which are significantly increased in the *tRNA-Ile-TAT-2–3* null cerebellum (Figures 7A, 7C, and 7D). Loss of *tRNA-Ile-TAT-1–1* reduced the tRNA^Ile^(TAT) pool to approximately 75% of that in wild-type mice (Figures 7A, 7C, and 7D).

The molecular changes caused by loss of *tRNA-Arg-TCT-4–1* are dramatically amplified in the absence of the ribosome rescue factors *Gtpbp1* or *Gtpbp2*, leading to severe neurodegeneration.^12,53^ This exacerbation of the phenotype caused by tRNA loss makes *Gtpbp2*^−/−^ mice an ideal genetic background to screen for the impact of tRNA mutations and identify vulnerable cell populations. We therefore crossed *tRNA-Ile-TAT-2–3*^−/−^ and *tRNA-Ile-TAT-1–1*^−/−^ mice to the sensitized *Gtpbp2*^−/−^ background. We found that homozygous loss of *tRNA-Ile-TAT-2–3*, which reduces the tRNA^Ile^ (TAT) pool in the cerebellum to 21% of that in wild-type mice, on the *Gtpbp2*^−/−^ background resulted in a significant loss of cerebellar granule cells in 12-week-old mice (Figures 7E and 7F). In contrast, granule cell survival was not impacted by mutation of *tRNA-Ile-TAT-1–1*, loss of which only depletes the tRNA^Ile^(TAT) pool by about 25%. To further elucidate the importance of the tRNA^Ile^(TAT) pool for cerebellar granule cell survival on a *Gtpbp2*^−/−^ background, we intercrossed *tRNA-Ile-TAT-2–3*^−/−^ and *tRNA-Ile-TAT-1–1*^−/−^ mice. We were unable to recover any double homozygous mutant mice at P14 (chi square, p value ≤ 0.01), indicating that expression of *tRNA-Ile-TAT-2–1* and *-2–2* alone are insufficient for viability. Heterozygosity for *tRNA-Ile-TAT-2–3* on a *tRNA-Ile-TAT-1–1* null background reduced the tRNA^Ile^(TAT) pool to approximately 32% of that in wild-type mice (Figures 7A and 7C). Surprisingly, despite this reduction, we did not observe cerebellar granule cell loss in these mice when crossed to a *Gtpbp2*^−/−^ background (Figures 7E and 7F). Heterozygosity for *tRNA-Ile-TAT-1–1* on a *tRNA-Ile-TAT-2–3* null background significantly reduced the compensatory upregulation of *tRNA-Ile-TAT-1–1*, further reducing the tRNA^Ile^(TAT) pool to approximately 11% of that in the cerebellum of wild-type mice (Figures 7A, 7C, and 7D). Heterozygous loss of *tRNA-Ile-TAT-1–1* on the *Gtpbp2*^−/−^, *tRNA-Ile-TAT-2–3*^−/−^ background significantly exacerbated cerebellar granule cell loss compared to that observed in *Gtpbp2*^−/−^, *tRNA-Ile-TAT-2–3*^−/−^ mice (Figures 7E and 7F). Together, our data indicates a steep cliff-like decline in cerebellar granule cell viability over a relatively narrow range of tRNA^Ile^(TAT) levels—that is, while a 68% drop in tRNA^Ile^(TAT) levels does not impact cerebellar granule cell survival, an additional 10%–20% decrease significantly impacts survival in a dose-dependent manner.

Surprisingly, the neurodegeneration induced by depletion of the tRNA^Ile^(TAT) family in *Gtpbp2*^−/−^ mice was less severe than that caused by the B6J mutation of *tRNA-Arg-TCT-4–1* (*n-Tr20* ^J/J^), despite the pronounced reduction in the tRNA^Ile^(TAT) pool in *tRNA-Ile-TAT-2–3*^−/−^, *tRNA-Ile-TAT-1–1*^+/−^ mice. Furthermore, neurodegeneration was completely restricted to cerebellar granule cells, in contrast to B6J-*Gtpbp2*^−/−^ mice, which have pronounced loss of dentate gyrus granule cells (Figure S8).^12,53^ This indicates that neurons are differentially vulnerable to the depletion of these two different tRNA isoacceptor families.

We hypothesized that the differential vulnerability could stem from differences in the available tRNA supply for decoding the respective cognate codons in the translatome. Indeed, comparing relative levels of the tRNA isoacceptor family measured by ChIP-seq and expression-weighted codon usage in the translatome determined by RiboTag mRNA sequencing, we found that the ratio of tRNA^Ile^(TAT) expression to the use of its cognate ATA codon in the translatome was almost double of that observed for the tRNA^Arg^(TCT) family to its cognate AGA codon (Figure 7G). This suggests that the tRNA^Ile^(TAT) family may be better buffered against insults by virtue of a more abundant supply of tRNA relative to its usage in the translatome. Thus, the impact of loss of tRNA genes on cellular homeostasis may depend on both their expression and contribution to their tRNA isoacceptor family, as well as the usage of their cognate codons in the translatome.

## DISCUSSION

Numerous neurological diseases have been linked to defects in tRNA biogenesis.^15^ However, our understanding of the mechanisms that underlie cell-type specificity in these disorders has been hampered by our inability to quantify tRNA expression in different cell types in a complex tissue like the brain. Here, we have developed a conditional epitope-tagged Pol III KI mouse, allowing us to leverage Pol III ChIP-seq to determine tRNA expression in discrete cell types in the nervous system.

We found that the expression of tRNA genes varies dramatically across nervous system cell types, though this frequently affects the composition, but not the levels, of tRNA isoacceptor families. While differential expression of tRNA isoacceptor families could modulate the translation of cognate codons, impacting ribosome decoding efficiency or speed,^34^ the biological consequence of differential tRNA isodecoder expression remains unclear. Sequence differences in the body of isodecoders may impact their translational efficiency,^54^ and thus, different isodecoder repertoires may serve to fine-tune translation. Growing evidence indicates that tRNAs can give rise to a variety of novel small non-coding RNAs.^55,56^ Indeed, a recent study proposed that the key function for differential isodecoder expression may be to give rise to different complements of tRNA fragments impacting diverse cellular pathways.^57^ For example, fragments derived from tRNA-Leu-CAG genes have been reported to regulate ribosome biogenesis,^58^ and members of this isoacceptor family have significant differences in expression between the various nervous system cell populations. Interestingly, a functional microRNA (miR-1983) is generated from the *tRNA-Ile-TAT-2–1* precursor,^59–62^ which we have shown is specifically expressed in a subset of neuronal populations. Of note, this microRNA is generated specifically from pre-*tRNA-Ile-TAT-2–1* and not *-2–2* or *-2–3*, which have identical mature sequences.

Intriguingly, we found that differential expression of tRNA isodecoders may underlie the vulnerability of certain neuronal populations to tRNA deficiency-induced ribosome stalling and neurodegeneration. For example, neuron populations that are vulnerable to mutation of *tRNA-Arg-TCT-4–1* have a more homogeneous tRNA^Arg^(TCT) pool relative to resilient populations such as Purkinje cells. Similarly, mutation of *tRNA-Ile-TAT-2–3* and *tRNA-Ile-TAT-1–1* specifically impacts cerebellar granule cell survival, while neuron populations with a more diverse tRNA^Ile^ (TAT) pool are spared. However, differential expression of individual tRNA genes is unlikely to be the sole factor determining cell-type vulnerability to tRNA mutations, and differences in usage of cognate codons or neuron-specific modulation of signaling pathways, such as mTOR, in response to ribosome stalling may also play a part.^53^

### Little is known about the mechanisms that regulate gene-specific expression of tRNAs

Multiple studies have reported that Pol II and Pol II-associated histone marks are found near tRNA genes with high Pol III occupancy, suggesting that active or open chromatin established by Pol II activity may gate Pol III access to tRNA genes.^63–66^ Indeed, chromatin accessibility at tRNA genes strongly correlates with tRNA abundances measured by Pol III ChIP-seq.^34^ However, the mechanisms governing Pol III transcription are more complex than mere regulation of chromatin accessibility. For instance, a recent study suggests that the presence of Pol II near tRNA genes can transcriptionally silence select tRNAs by directly modulating Pol III in a gene-specific manner.^67^ In addition, Pol II TFs such as SOX4, have been reported to directly inhibit TFIIIB binding and therefore Pol III recruitment to certain tRNA genes in human glioblastoma cells.^68^ Numerous Pol II TF binding motifs are found near tRNA genes, and binding of canonical Pol II TFs at these sites could alter recruitment of Pol III transcriptional machinery, proving an intriguing mechanism for dynamic cell-type-specific regulation of tRNA genes.^63,65,69^ The molecular players and mechanisms that result in cell-type-specific tRNA expression may be shared between subsets of tRNA genes (for instance, those that are significantly upregulated in cerebellar granule cells relative to other neuronal populations) or may be as varied as the genes themselves. Our data provide an essential platform to investigate the cell-type-specific regulation of tRNA expression.

## STAR★METHODS

### RESOURCE AVAILABILITY

#### Lead contact

Further information and requests for resources and reagents should be directed to and will be fulfilled by the lead contact, Susan Ackerman (sackerman@health.ucsd.edu).

#### Materials availability

Mouse lines generated in this study are available on request to the lead contact.

#### Data and code availability

The datasets generated during this study have been deposited at GEO: GSE239759 and GEO: GSE250090 and will be publicly available as of the date of the publication. This paper does not report any original code.

### EXPERIMENTAL MODEL AND STUDY PARTICIPANT DETAILS

#### Mouse husbandry

All mouse studies were performed under the guidance of the University of California, San Diego in accordance with institutional and regulatory guidelines. Mice were group-housed in a 12-h light/dark cycle with free access to food and water. Mice from both sexes were used for all experiments and animal ages are specified in the respective Methods or Results.

#### Mouse strains

The Polr3a-cKI targeting construct was generated by recombineering and contained a ~800-nt homology arm, a *loxP* site upstream of exon 31, a 3X-stop sequence downstream of exon 31, a Frt-flanked *Neo* resistance cassette (driven by the PGK promoter), a *loxP* site, a 3X FLAG-tagged exon 31, and a ~800-nt homology arm.^79^ Targeted B6(Cg)-*Tyr*^*c-2J*^/J ES cells used to make Polr3a-cKI mice were generated by electroporation of the *Polr3a* targeting construct, Cas9 protein and gRNA (TTGCCCTTGTATTCGTACAC AGG) into (B6(Cg)-Tyr^c–2J^/J (Jax #000058). G418-resistant ES clones were screened for correct targeting by nested PCR using primers outside the construct and primers inside the *neo* cassette. Three positive ES clones were aggregated with 8-cell C57BL6/J embryos to generate chimeric mice. Chimeric mice harboring the knock-in cassette were selected by coat color, confirmed by PCR, and tested for germline transmission. The *neo* cassette was removed by breeding confirmed germline animals with B6J.129S4-Gt(ROSA)26sor-tm1(FLP1)Dym/J (Janelia Research Campus) homozygous females (Figure S1B).

Generation of the B6J-Tg(*tRNA-Arg-TCT-3–1*)/*n-Tr21* and B6J-Tg(*tRNA-Arg-TCT-1–1*)/*n-Tr22* transgenic mouse lines, B6J-*tRNA-Arg-TCT-4–1*^−/−^ (*n-Tr20*) knockout mouse line, the B6J.B6N congenic mouse line in which the wild-type *tRNA-Arg-TCT-4–1* allele has been transferred from B6N mice onto the B6J background, the B6J.B6N-*tRNA-Ile-TAT-2–3*^−/−^ (*n-Ti17*) mutant mouse line, and the B6J-*Gtpbp2*^−/−^ mouse line have been previously described.^7,12,70^ B6J.B6N-*tRNA-Ile-TAT-1–1*^−/−^ (*n-Ti16*) mice were generated at The Jackson Laboratory using the same targeting strategy that had previously been used to create the B6J.B6N-*tRNA-Ile-TAT-2–3*^−/−^ mouse line.^7^ Briefly, *tRNA-Ile-TAT-1–1* was targeted using CRISPR-Cas9 via pronuclear injection of B6J fertilized oocytes with Cas9 mRNA and single-guide RNAs (sgRNA) targeting the 5′ region of the tRNA. The injected zygotes were transferred into pseudo-pregnant female mice to obtain live pups. The offspring were screened for deletions within *tRNA-Ile-TAT-1–1*, and a founder with a 15 bp deletion within the 5′ exon of *tRNA-Ile-TAT-1–1* was selected (a mutation identical to that generated in *tRNA-Ile-TAT-2–3*) and crossed to congenic B6J.B6N mice that carry the wild-type *tRNA-Arg-TCT-4–1* gene. *B6N-tRNA-Arg-TCT-1–1*^−/−^ mice were generated at The Jackson Laboratory using CRISPR-Cas9 via pronuclear injection of B6N fertilized oocytes with Cas9 mRNA and a pair of guide RNAs flanking the tRNA gene. The injected zygotes were transferred into pseudo-pregnant female mice to obtain live pups. The offspring were screened for deletions within *tRNA-Arg-TCT-1–1* locus, and a founder with a 93-nucleotide deletion that completely removed the *tRNA-Arg-TCT-1–1* gene was selected and backcrossed to congenic B6J.B6N mice for 10 generations. B6N.129-*Rpl22*^*tm1.1Psam*^/J mice (carrying the RiboTag mutation of the ribosomal protein L22 locus) were obtained from The Jackson Laboratory.

For cell-type-specific tRNA and codon usage datasets, Cre-lines were used and the Cre was paternally inherited to generate conditionally targeted cell populations (see Table S2). B6.129-Tg(Pcp2-cre)2Mpin/J (JAX#004146),^80^ B6; FVB-Tg(Aldh1l1-cre/ERT2)1Khakh/J (JAX#029655),^81^ C57BL/6-Tmem119^em1(cre/ERT2)Gfng/J^ (JAX#031820),^82^ B6.129S-ChAT^tm1(cre del–Neo)Lowl^/MwarJ (JAX#031661),^83,84^ B6.SJL-Slc6a3^tm1.1(cre)Bkmn/J^ (JAX#006302),^85^ B6.Cg-Tg(Plp1-Cre/ERT)3Pop/J (JAX#005975),^86^ B6.Cg-Tg(Pdgfrb-Cre/ERT2)6096Rha/J (JAX#029684),^87,88^ B6N.Tg(Dlx6a-cre)1Mekk/J (JAX#008199),^89,90^ and B6N.Cg^Tg(Sox2–CRE)1Amc^/J (JAX#014094)^91^ were obtained from The Jackson Laboratory. B6.FVB(Cg)-Tg(Rbp4-cre)KL100Gsat/Mmucd was obtained from MMRRC-UCD (#037128).^48,92–94^ B6.Tg(Gabra6-Cre)B1Lfr mice are as previously published.^71^ For inducible Cre-lines, tamoxifen (Sigma-Aldrich, T5648) was dissolved in corn oil (C8267) at a concentration of 10 mg/ml and administered by oral gavage at 75 mg/kg body weight on 5 consecutive days beginning at 3 weeks of age. Induction of Cre activity by tamoxifen and cell type specificity was confirmed by crossing all CreERT lines to the TdTomato B6.Cg-Gt(ROSA)26Sor^tm14(CAG-tdTomato)Hze/J^ (Jax#007914) reporter mouse line (Ai14).

### METHOD DETAILS

#### Northern blot analysis

RNA extraction and northern blot analysis was performed as previously described.^7,12^ In brief, total RNA was extracted from tissue from 6–8-week-old mice using TRIzol reagent (Invitrogen), resolved on denaturing 15% urea-acrylamide gels, and blotted onto Hybond-N+ membranes (GE Healthcare Life Sciences). The membranes were hybridized with 5′ ^32^P-labeled oligonucleotide probes at 55°C in hybridization buffer containing 6x SSC, 0.01 M sodium phosphate, 1 mM EDTA, 0.25% SDS and 100 μg/mL salmon sperm DNA. When increased stringency was required (as for the probes distinguishing *tRNA-Ile-TAT-2* from *tRNA-Ile-TAT-1–1* or for pooled probes), hybridization was performed at 60°C in hybridization buffer containing 3M TMACl rather than 6x SSC.

The four members of the tRNA^Ile^(TAT) family have unique introns and thus the expression of the immature or precursor tRNAs was assessed using probes that recognized their unique intron sequences (Table S5). Expression of the mature tRNA^Ile^(TAT) genes was detected using three different probes: one that recognized the three identical *tRNA-Ile-TAT-2-(1,2,3)* genes, but not *tRNA-Ile-TAT-1–1*; a second that recognized all four members of the tRNA^Ile^(TAT) family; and a third that specifically recognized *tRNA-Ile-TAT-1–1*.

#### ChIP-seq library preparation

ChIP-seq libraries were generated from target tissues containing conditionally epitope-tagged cell populations from 6-week-old mice (Table S2). Crosslinking and library generation was performed as described in^95^ with some modifications. In brief, extracted tissue was diced with a cold razor blade and crosslinked in 1% formaldehyde for 10 min at room temperature. Crosslinking was terminated with 1/20^th^ volume of 2.625M glycine, washed with PBS containing protease inhibitors, and samples were homogenized in ChIP RIPA buffer (50 mM Tris-Hcl pH 7.5, 150 mM NaCl, 1 mM EDTA, 1% IGEPAL CA-630, 0.25% Na-Deoxycholate, 0.1% SDS, with cOmplete mini protease inhibitor cocktail (Roche)). Approximately 400–600 μL of crosslinked lysate was fragmented using a Branson Digital Sonifier Probe (12–14x cycles, 40% amplitude, 10 s ON, 30 s OFF). Samples were centrifuged for 10 min at 4°C and the lysate was transferred to a new tube. 1% of the sample was stored at 20°C for DNA input control for each sample. For ChIP, 20 μL Protein G Dynabeads and 2 μg of FLAG antibody (Sigma-Aldrich, F1804) were added to cleared lysate and rotated overnight at 4°C. Dynabeads were washed 3 times with Wash Buffer 1 (20 mM Tris-HCl pH 7.4, 150 mM NaCl, 2 mM EDTA, 0.1% SDS, 1% Triton X-100), three times with Wash Buffer 2 (250 mM LiCl, 1 mM EDTA, 1% Triton X-100, 0.7% sodium deoxycholate), three times with TET (10 mM Tris-HCl pH 8, 1 mM EDTA, 0.2% Tween 20), and resuspended in nuclease-free water.

ChIP DNA from ChIP input and ChIP samples was used to prepare libraries using the NEBNext Ultra II Library preparation kit (NEB E7645S) and KAPA Single-Indexed Adapters for Illumina (Roche, KR1317) according to manufacturer’s protocol. Libraries were amplified by PCR for 14 cycles, size selected for 150–500 bp fragments and sequenced, yielding ~10–40 million reads per sample. A minimum of two biological replicates were generated for each cell type.

#### RiboTag RNA extraction and library construction

Female B6N.129-*Rpl22*^*tm1.1Psam*^/J mice were crossed to the appropriate Cre driver lines (Table S2) to generate *Rpl22* ^fl/+^, Cre^Tg/+^ mice. The appropriate brain region (granule cells: cerebellum; astrocytes, microglia, and layer V cortical neurons: cortex; inhibitory interneurons and dentate gyrus cells: hippocampus) was isolated from 6-week-old mice. For isolation of ribosome associated RNA from inhibitory interneurons (Dlx6a-Cre), hippocampi from 2 mice were pooled for each biological replicate. Three biological replicates were generated for each genotype.

Freshly isolated tissue was homogenized on ice using a Dounce homogenizer in lysis buffer (50 mM Tris-Cl pH 7.5, 100 mM KCl, 12 mM MgCl_2_, 1mM DTT, 100 μg/ml cycloheximide, 1% (v/v) NP-40, 1 mg/ml heparin, 25 units/ml Turbo DNaseI (ThermoFisher Scientific), 20 units/ml Superase-In RNAse inhibitor (ThermoFisher Scientific), cOmplete mini protease inhibitor cocktail (Roche)). Ribosomes were then isolated as previously described, with some minor modifications.^43^ Briefly, for each sample, 200 μL of Pierce anti-HA magnetic beads (ThermoFisher Scientific) was washed in lysis buffer and then added to the lysate containing tagged ribosomes. The samples were incubated for 4 h at 4°C with rotation. Ribosome-bound beads were then washed 3 times for 5 min each in 1 mL of high salt wash buffer (50 mM Tris pH 7.4, 300 mM KCl, 12 mM MgCl_2_, 0.5 mM DTT, 100 μg/ml cycloheximide, 1% (v/v) NP-40, 20 units/ml Superase-In RNAse inhibitor, cOmplete mini protease inhibitor cocktail). After the final wash, the ribosome-bound RNA was eluted using buffer RLT (supplemented with b-mercaptoethanol) and isolated using the Qiagen RNeasy mini kit following manufacturer’s instructions. RNA was eluted in 30 μL nuclease-free H_2_O and the quality and concentration were assessed using high sensitivity RNA ScreenTape on a TapeStation (Agilent) and the Qubit RNA high sensitivity kit on a Qubit 2.0 fluorometer (Life Technologies).

Three mRNA libraries were generated for each genotype using the Kapa Stranded mRNA-Seq Kit (Roche) according to the manufacturer’s protocol. Paired-end reads (2× 100 bp) were obtained using the NovaSeq 6000 sequencer (Illumina).

#### Histology

Anesthetized mice were transcardically perfused with Bouin’s fixative. Brains were post-fixed overnight and embedded in paraffin. Sections were deparaffinized, rehydrated, and stained with hematoxylin and eosin according to standard protocols. Histological slides were imaged using a digital slide scanner (Hamamatsu).

For quantification of cerebellar granule cells, the total number of granule cells (viable and pyknotic) were counted in a 0.0125 mm^2^ area from lobule III and averaged from three sections per brain spaced 100 μm apart at midline. All histological quantifications were performed with three to four mice of each genotype using mice of both sexes at 12 weeks of age, except for B6J-*Gtpbp2*^−/−^ mice which were analyzed at 8 weeks of age.

#### Western blot analysis

Proteins were extracted from relevant tissues of 6-week-old mice homogenized in 5 volumes of cold lysis buffer (50 mM Tris-HCl pH 8.0, 150 mM NaCl, 1% Triton X-100, 0.5% sodium deoxycholate, 0.1% SDS) supplemented with phosphatase and protease inhibitors (PhosStop and cOmplete Mini, EDTA-free Protease inhibitor Cocktail, Roche). Proteins were resolved on SDS-PAGE gels prior to transfer to PVDF membranes. Western blotting was performed according to standard protocols. Primary antibodies used: rabbit anti-FLAG (Cell Signaling Technologies D6W5B) and mouse anti-vinculin (Sigma V9131). Signals were detected with SuperSignal West Pico PLUS Chemiluminescent substrate.

### QUANTIFICATION AND STATISTICAL ANALYSIS

#### ChIP-seq analysis

ChIP-seq reads were mapped to the mm10 genome using bowtie2^75^ with the following parameters: “bowtie2 -q –very-sensitive-local -N 1”. Peaks representing areas of strong polymerase binding were called using HOMER^72^ with the following parameters: “-style factor -fragLength 150”.

tRNA binding was quantified by counting the number of reads that overlapped tRNA genes with discrete anticodons (n = 465) using HOMER’s annotatePeaks function. The tRNA file (mm10-tRNAs.bed) was obtained from the GRC38/mm10 build of the gtRNAdb.^2^ tRNAs with ambiguous anticodons or amino acids, that is, genes annotated as tRX, were omitted from the analysis, yielding 465 tRNA genes. The high-confidence tRNA gene set (401 genes) was obtained from the mm10-tRNAs-confidence-set.out file. tRNA genes were classified as expressed or occupied if they had >2 reads overlapping the tRNA gene locus in both biological replicates. We calculated the relative expression of the 49 tRNA anticodon families by normalizing the sum of the Pol III ChIP-seq reads mapping to all the tRNAs of a given isoacceptor family to the total number of reads mapping to all tRNA genes. The relative levels of tRNA isotypes were determined by normalizing the sum of the Pol III ChIP-seq reads mapping to all the tRNAs that encode the same isotype to the total number of reads mapping to all tRNA genes.

For comparison of tRNA abundance measurements inferred from our Pol III ChIP-Seq datasets with data obtained from QuantM-tRNA Seq (Quantitative Mature tRNA sequencing),^6^ we simplified our data by summing reads that were assigned to tRNAs with identical mature sequences (for example, *tRNA-Ile-TAT-2–1*, *2–2*, and *2–3*) to assess expression of the 273 distinguishable tRNA genes.

The following publicly available ChIP-seq datasets were analyzed: adult liver (ArrayExpress: E-MTAB-424, GEO: GSE33421, and ArrayExpress: E-MTAB-2326),^5,8,22^ embryonic (E15.5) brain and liver (ArrayExpress: E-MTAB-2326),^5^ mouse embryonic stem cells and mouse embryonic fibroblasts (GEO: GSE143969 and ArrayExpress: E-MTAB-767),^23,27^ and mouse hepatocarcinoma cell lines (GEO: GSE47849 and ArrayExpress: E-MTAB-4046).^26,28^ For Table S3, tRNA genes were classified as expressed based on criteria proposed by the initial study, wherever possible. For POLR3D ChIP-Seq from Hepa1–6 cells,^28^ reads overlapping with tRNAs were first normalized to reads in the input sample.

For differential analysis of tRNA expression between two cell populations, the variances of the populations for each tRNA gene, isoacceptor family, or isotype were checked using an F-test, and based on its results, either a Student’s or Welch’s t test was performed, followed by a Benjamini-Hochberg correction for multiple comparison. Comparisons with a Benjamini-Hochberg corrected p value (q value) ≤ 0.05 were considered significant.

For analysis of high-variance tRNAs, we filtered out tRNAs where the variance within a cell type was greater than the variance of the population as a whole.

#### RiboTag RNA-seq data analysis

Reads were trimmed to remove adapter sequences (AGATCGGAAGAG) using cutadapt version 3.5^77^ and then quantified using kallisto version 0.46.2^73^ pseudo-aligning to a Gencode M24 transcript reference. Differential expression was performed using sleuth version 0.30.0,^74^ accounting for batch covariates when applicable.

To calculate the codon usage across cell types, we weighted the frequencies of the 61 sense codons of each protein-coding transcript by their cell type-specific expression (transcripts per million, tpm). The overall usage of each codon in a cell type was obtained by summing these values across all expressed transcripts. Briefly, the codon content for each protein-coding transcript was acquired from Ensembl^76^ (GRCm38.p6) using biomartr version 1.0.2.^78^ If multiple peptides were annotated for a single transcript, the longest annotated peptide was kept. Analysis was limited to transcripts in the GENCODE basic set, i.e., transcripts with full-length CDS. The number of occurrences of each sense codon was multiplied by the transcript expression (tpm, determined by kallisto) to determine the expression-weighted use of the codon. Next, the overall usage of the codon for each library was obtained by summing the values across all transcripts. Relative codon usage was calculated as the usage of the codon of interest normalized to the sum of the usage of all 61 sense codons and is expressed as a percentage of total use.

To eliminate the possibility that differences in codon usage in cell-type-specific genes arose simply from stochastic variation in codon usage because of smaller gene set sizes, we calculated empirical p values using permutation tests. Briefly, to determine if the Pearson correlation between cell-type-specific genes was lower than expected by chance, we randomly sampled the appropriate set sizes (that is, the number of genes that were 50-fold upregulated in cortical neurons relative to astrocytes, and vice-versa) from the entire translatome 10,000 times and calculated the Pearson correlation between these sets to empirically determine the p value.

Similarly, to determine whether gene set size was responsible for differences in codon usage observed between transcripts that were differentially expressed between two populations, we sampled random sets of genes and calculated the expression weighted codon usage of these sets in the appropriate cell type. For each set size, we created 10,000 random gene sets from the ~27,000 detected protein-coding transcripts. For each set, we calculated the weighted codon usage as previously explained. From this, we determined if the usage of a particular codon in each gene set diverged more significantly from that of the general translatome than expected by chance, i.e., if it was significantly higher or lower than that expected. We calculated two empirical p values defined as the fraction of random gene sets of the same size whose usage of the codon of interest was either higher or lower than that of the gene set of interest. We defined gene sets with p ≤ 0.05 as having a codon usage that diverges more from the translatome than expected by chance.

Purkinje cell translatome data were previously generated from 3-week-old mice^44^ and are publicly available at GEO: GSE223881.

#### Statistics

Graphs were generated and statistical analysis was performed in GraphPad Prism 9 and using custom R scripts. Heatmaps were generated using R package pheatmap using standard settings. Cell types were clustered by Euclidean distance, and dendograms were sorted and ordered using R package dendsort. Principal component analysis was performed, and confidence ellipses were generated R packages FactoMineR and factoextra using standard settings. The proportion of variance explained by each principal component is indicated on PCA plots.

tRNA distribution histograms and curves were generated using Prism 9 using the frequency distribution function, followed by nonlinear regression (curve-fit, Gaussian). Statistical parameters including the value of n and statistical significance are reported in the figures and figure legends. For northern blot analyses, n represents individual mice, and the individual datapoints are shown in the figure.

## Supplementary Material

MMC6

MMC5

MMC3

MMC4

MMC2

MMC1

## Figures and Tables

**KEY RESOURCES TABLE:** 

REAGENT or RESOURCE	SOURCE	IDENTIFIER

Antibodies		

Mouse anti-FLAG (M2)	Sigma-Aldrich	Cat #F1804; RRID: AB_262044
Rabbit anti-DYKDDDK	Cell Signaling Technologies	Cat #14793; RRID: AB_2572291
Mouse anti-vinculin	Sigma-Aldrich	Cat #V9131; RRID: AB_477629
Pierce Anti-HA Magnetic Beads	ThermoFisher Scientific	Cat #88836; RRID: AB_2861399

Chemicals, peptides, and recombinant proteins		

Tamoxifen	Sigma-Aldrich	Cat #T5648

Critical commercial assays		

NEBNext Ultra II DNA library preparation kit	New England Biolabs	Cat #E7103
KAPA Single-Indexed Adapters (Illumina Platforms)	Roche	Cat #KR1317
KAPA Stranded mRNA-Seq Kit (Illumina Platforms)	Roche	Cat #KR0960
RNeasy Mini Kit	Qiagen	Cat #74104

Deposited data		

RiboTag RNA-Seq	This study	GEO: GSE239759
Pol III (Flag) ChIP-Seq	This study	GEO: GSE250090
POLR3A ChIP-Seq (adult liver, E15.5 brain and liver)	Schmitt et al.^5^	ArrayExpress: E-MTAB-2326
POLR3A ChIP-Seq (adult liver)	Kutter et al.^8^	ArrayExpress: E-MTAB-424
POLR3A ChIP-Seq (adult liver)	Canella et al.^22^	GEO: GSE33421
POLR3C ChIP-Seq (mESC, MEFs)	Wang et al.^27^	GEO: GSE143969
POLR3A and POLR3C ChIP-Seq (mESCs)	Carriere et al., 2012^23^	ArrayExpress: E-MTAB-767
POLR3D ChIP-Seq (Hepa 1–6 cells)	Renaud et al.^28^	GEO: GSE47849
POLR3A ChIP-Seq (Hepa1c1c7 cells and Hepa 1–6 cells)	Rudolph et al.^26^	ArrayExpress: E-MTAB-4046
Purkinje cell, RiboTag RNA-Seq (3-week-old mice)	Griffin et al.^44^	GEO: GSE223881

Experimental models: Organisms/strains		

Polr3a-cKI	This study	N/A
B6J-*Gtpbp2^−/−^* (C57BL/6J-*Gtpbp2^nmf205^*/J)	Ishimura et al.^12^	RRID: IMSR_JAX:004823
B6J.B6N^*Arg-TCT-4-1/nTr20*^	Ishimura et al.^12^	N/A
B6J-*Arg-TCT-4-1(nTr20)* ^−/−^	Ishimura et al.^70^	N/A
B6J-Tg(*Arg-TCT-1-1/n-Tr22*)529	Kapur et al.^7^	N/A
B6J-Tg(*Arg-TCT-3-1*)516	Kapur et al.^7^	N/A
B6J.N-*Ile-TAT-2-3(nTi17)*^−/−^	Kapur et al.^7^	N/A
B6J.N-*Ile-TAT-1-1(nTi16)*^−/−^	This study	N/A
B6J.N-*Arg-TCT-1-1(nTr22)* ^−/−^	This study	N/A
B6.Cg-*Gt(ROSA)26Sor^tm14<CAG-tdT^°^mat^°^)Hze^*/J	The Jackson Laboratory	RRID: IMSR_JAX:007914
B6.129-Tg(Pcp2-cre)2Mpin/J	The Jackson Laboratory	RRID: IMSR_JAX#004146
B6; FVB-Tg(Aldh1l1-cre/ERT2)1 Khakh/J	The Jackson Laboratory	RRID: IMSR_JAX#029655
C57BL/6-Tmem119em1(cre/ERT2)Gfng/J	The Jackson Laboratory	RRID: IMSR_JAX#031820
B6.129S-ChAT^tm1(cre del–Neo)Lowl^/MwarJ	The Jackson Laboratory	RRID: IMSR_JAX#031661
B6.SJL-Slc6a3^tm11(cre)Bkmn/J^	The Jackson Laboratory	RRID: IMSR_JAX#006302
B6.Cg-Tg(Plp1-Cre/ERT)3Pop/J	The Jackson Laboratory	RRID: IMSR_JAX#005975
B6.Cg-Tg(Pdgfrβ-Cre/ERT2)6096Rha/J	The Jackson Laboratory	RRID: IMSR_JAX#029684
B6N.Tg(Dlx6a-cre)1Mekk/J	The Jackson Laboratory	RRID: IMSR_JAX#008199
B6N Cg^Tg(Sox2–CRE)1Amc/J^	The Jackson Laboratory	RRID: IMSR_JAX#014094
B6.FVB(Cg)-Tg(Rbp4-cre)KL100Gsat/Mmucd	The Jackson Laboratory	RRID: IMSR_MMRRC-UCD#037128
B6.Tg(Gabra6-Cre)B1Lfr	Fünfschilling et al.^71^	N/A
B6J.129S4-Gt(ROSA)26sor-^tm1(FLP1)Dym^/J	Janelia Research Campus	N/A
B6J.129(Cg)-*Rpl22^tm,,Psam^*/SjJ	The Jackson Laboratory	RRID: IMSR_JAX:029977

Oligonucleotides		

*Polr3a* guide RNA: TTGCCCTTGTATTCGTACACAGG	This study	N/A
For northern blot probe sequences, please see Table S5		N/A

Software and algorithms		

HOMER	Heinz et al.^72^	RRID: SCR_010881; http://homer.ucsd.edu
kallisto vO.46.2	Bray et al.^73^	RRID: SCR_016582; https://pachterlab.github.io/kallisto/about
sleuth vO.30.0	Pimentel et al.^74^	RRID: SCR_016883; https://pachterlab.github.io/sleuth/about
bowtie2 v 2.2.3	Langmead and Salzberg^75^	RRID: SCR_016368; http://bowtie-bio.sourceforge.net/bowtie2/index.shtml
ensembldb v2.6.8	Rainer et al.^76^	RRID: SCR_019103; https://www.bioconductor.org/packages/release/bioc/html/ensembldb.html
Cutadapt v3.5	Martin et al.^77^	RRID: SCR_011841; http://code.google.com/p/cutadapt/
Biomartr v1.0.2	Drost et al.^78^	https://cran.r-project.org/web/packages/biomartr/index.html
pheatmap		RRID: SCR_016418; https://www.rdocumentation.org/packages/pheatmap/versions/0.2/topics/pheatmap
factoextra		RRID: SCR_016692; https://cran.r-project.org/web/packages/factoextra/index.html
FactoMineR		RRID: SCR_014602; http://factominer.free.fr/index.html
dendsort		RRID: SCR_016693; https://cran.r-project.org/web/packages/dendsort/index.html
GraphPad Prism v9		RRID: SCR_002798

## Highlights

- Conditional epitope-tagged Pol III mouse reveals cell-type-specific tRNA expression
- tRNA gene expression varies dramatically between different neural cell populations
- Neurons are differentially vulnerable to insults in distinct tRNA isoacceptor families
- Heterogeneity in tRNA pools contributes to resilience to tRNA mutation or loss
