# Supplementary material for: Cell-type-specific expression of tRNAs in the brain regulates cellular homeostasis
Source: Neuron. Author manuscript; Available in PMC 2024 May 3. (PMC11065635; doi:10.1016/j.neuron.2024.01.028)
Supplement: MMC1 [file NIHMS1969755-supplement-MMC1.pdf]

Neuron, Volume *112*

Supplemental information

Cell-type-specific expression of tRNAs
in the brain regulates cellular homeostasis

Mridu Kapur, Michael J. Molumby, Carlos Guzman, Sven Heinz, and Susan L. Ackerman

Supplementary Figure S1

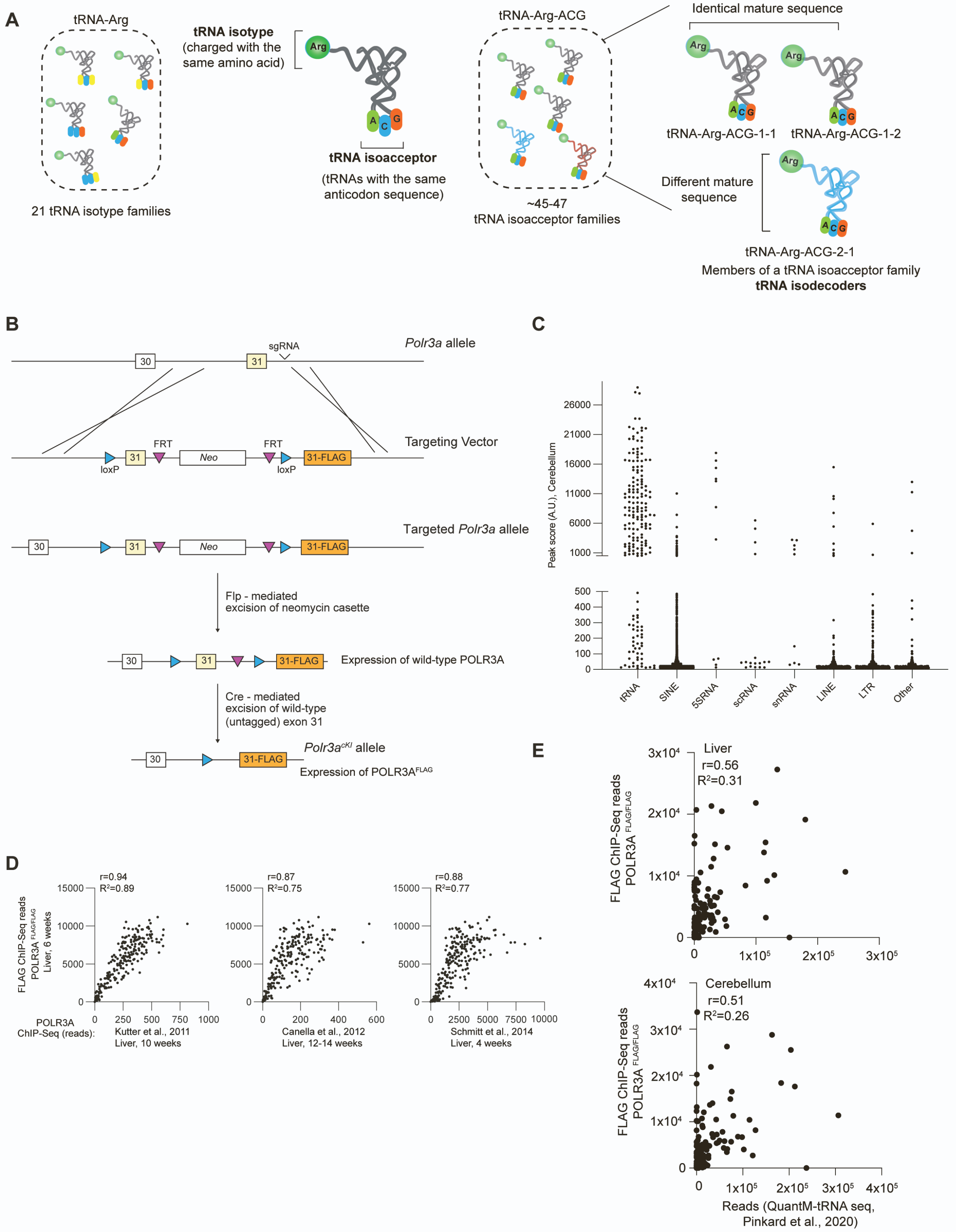

Figure S1. Generation and validation of the conditional epitope-tagged *Polr3a-cKI* mouse, related to Figure 1

(A) tRNA nomenclature. tRNAs charged with the same amino acid belong to the same isotype. There are 21 isotype families, including $tRNA^{Sec}$ (selenocysteine). An isotype family can contain tRNAs that are charged by the same amino acid but have different anticodons (isoacceptors). tRNAs with the same anticodon are members of a tRNA isoacceptor family. There are approximately 45 tRNA isoacceptor families in mice. Members of an isoacceptor family may have identical mature sequences, or differences in the tRNA body (isodecoders). tRNAs are named based on their isotype, anticodon, transcript ID, and gene locus ID (*tRNA-isotype-anticodon-transcript ID-gene locus ID*). tRNAs with identical mature sequences have the same transcript ID, but different gene locus IDs.

(B) Schematic depicting the targeting and generation of the conditional *Polr3a-cKI* mouse. The *Polr3a-cKI* allele was generated by CRISPR/Cas9-mediated homologous recombination. The targeting allele contains a *loxP* site upstream of exon 31 of *Polr3a*, followed by a neomycin cassette flanked by FRT sites, a *loxP* site, and then a 3x-FLAG-tagged exon 31. Flippase (Flp)-mediated excision of the *neomycin* cassette (*Neo*) was used to generate the conditional *Polr3a-cKI* allele. Cre-mediated recombination results in the replacement of wild-type (untagged) exon 31 with the FLAG-tagged exon 31, resulting in the incorporation of the FLAG-tag on the C-terminus of POLR3A.

(C) POLR3A-FLAG occupancy peak score distribution in mouse cerebellum, separated by gene annotation. Each point is a detected peak.

(D) Scatter plots comparing POLR3A^{FLAG} ChIP-Seq reads to published endogenous Pol III (POLR3A) ChIP-Seq data sets for individual tRNA genes from the mouse liver. The age of the mice used for library generation is indicated. Pearson (r) correlation coefficients and coefficients of determination (R^2) are shown.

(E) Scatter plots comparing reads mapping to tRNA genes in mouse liver (above) and cerebellum (below) between POLR3A^{FLAG} ChIP-Seq and QuantM-tRNA seq. ChIP-Seq reads for identical tRNA genes were pooled for comparison. Pearson (r) correlation coefficients and coefficients of determination (R^2) are shown.

Supplementary Figure S2

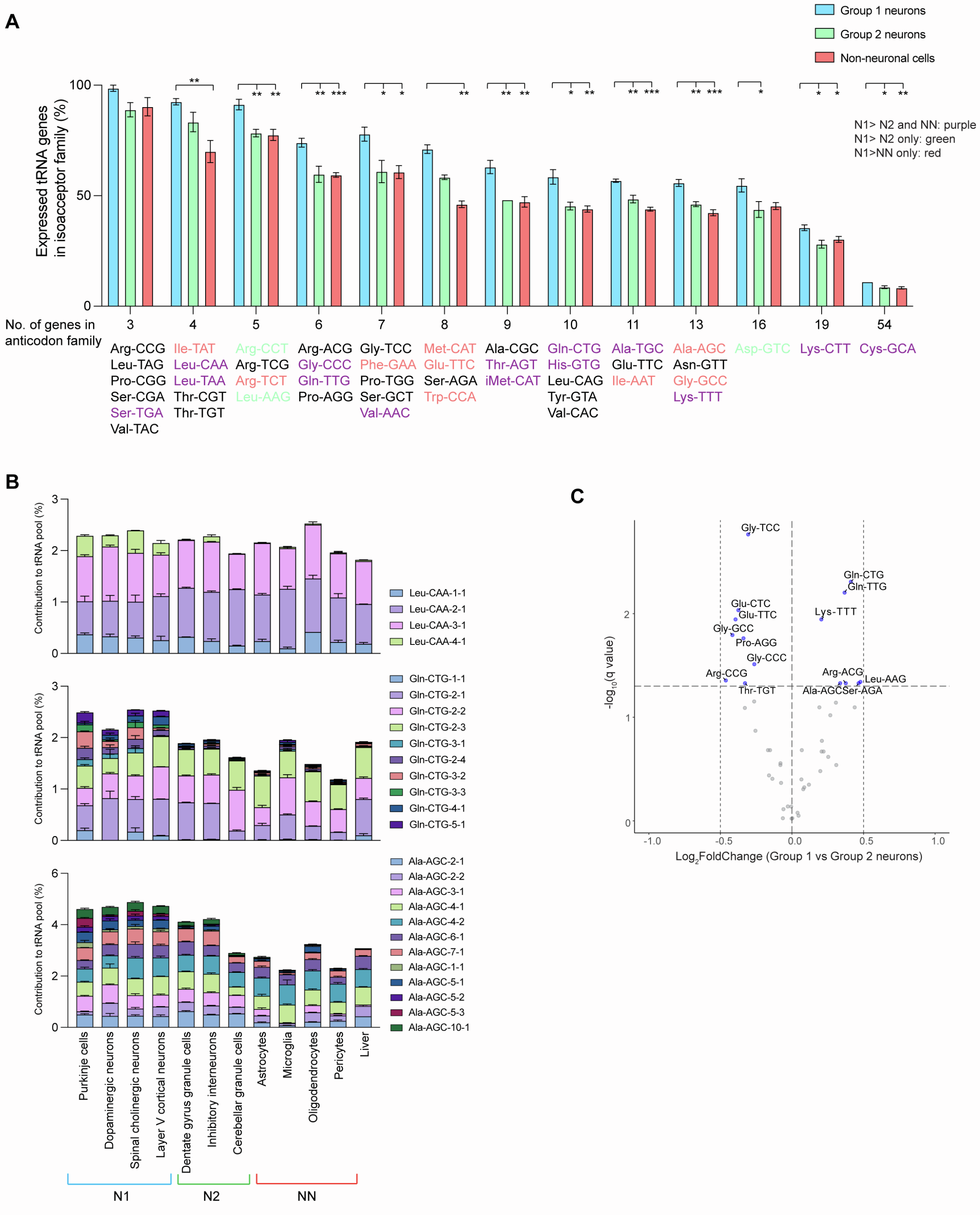

Figure S2. Increased diversity in the composition of tRNA isoacceptor families in Group 1 neurons, related to Figure 2

(A) The percentage of expressed tRNA genes (which make up >5% of the tRNA isoacceptor family) in tRNA isoacceptor families, grouped by family size. The tRNA families are listed below. tRNA families for which group 1 neurons (N1) express a significantly higher percentage of tRNA genes relative to both group 2 neurons (N2) and non-neuronal cells (NN) are labeled in purple (N1 > N2 and NN). tRNA families for which group 1 neurons express a higher percentage of tRNA genes relative to non-neuronal cells (N1 > NN) or group 2 neurons (N1 > N2) are labeled in red and green respectively. One-way ANOVA with Tukey post-test. * $p \leq 0.05$, ** $p \leq 0.01$, *** $p \leq 0.001$, **** $p \leq 0.0001$

(B) Composition of tRNA isoacceptor families with increased diversity in group 1 (N1) neurons relative to group 2 neurons (N2) and/or non-neuronal (NN) cells. The $tRNA^{Leu}$(CAA), $tRNA^{Gln}$(CTG), and $tRNA^{Ala}$(AGC) families are shown. The relative contributions of individual members of tRNA families to the global tRNA pool (mean + SEM) are shown in the indicated colors. N1: group 1 neurons, N2: group 2 neurons, NN: non-neuronal cells.

(C) Differentially expressed tRNA isoacceptor families between group 1 and group 2 neuronal populations. All tRNA isoacceptor families with q value ≤0.05 are highlighted in

blue and labeled. Horizontal dashed line: q value = 0.05. Vertical dashed lines: $Log_2FoldChange$ = 0.5 and -0.5.

Supplementary Figure S3

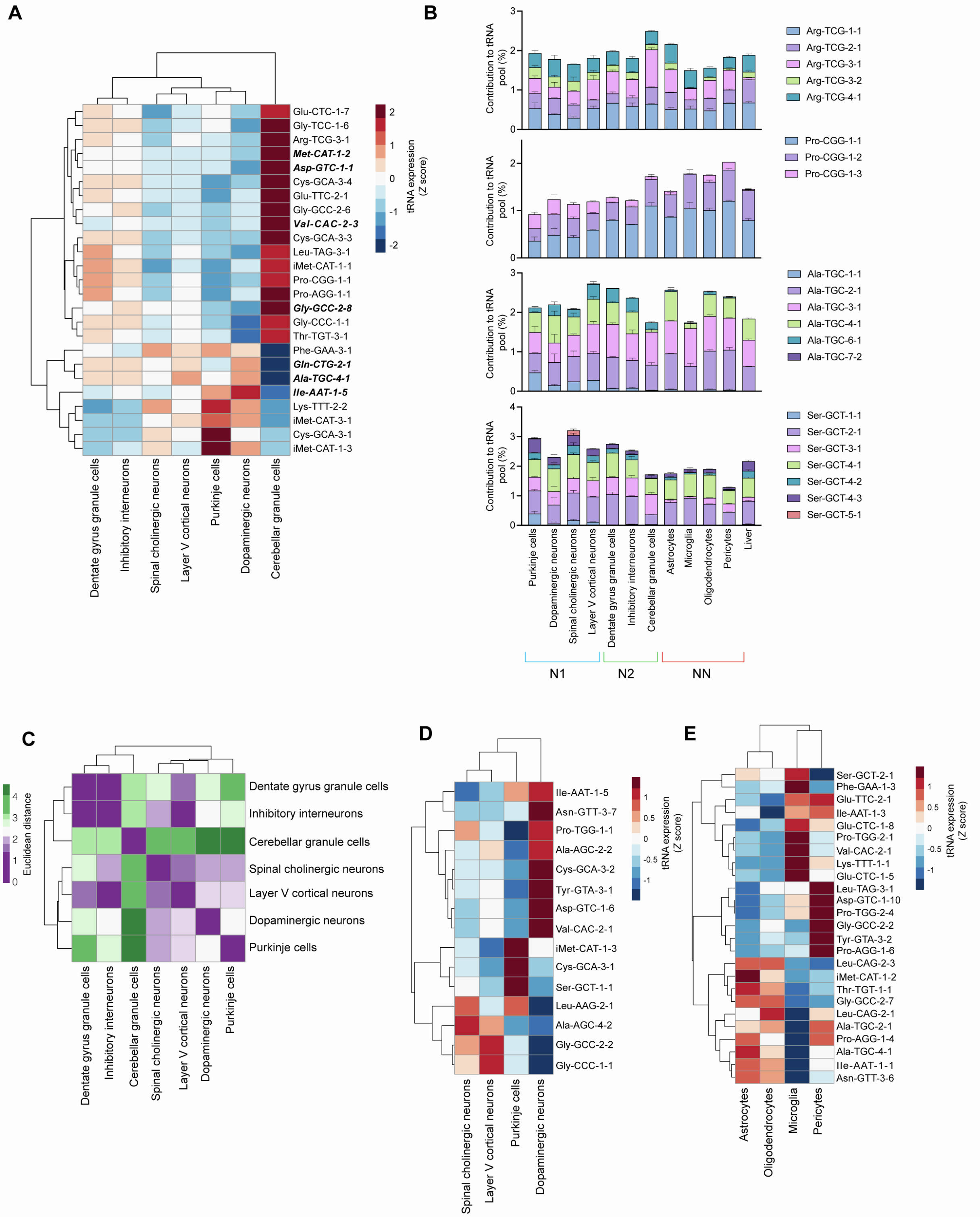

Figure S3. Variation of tRNA expression across cell types in the nervous system, related to Figure 2

(A) Heatmap of the *Z* scores for expression of the 25 tRNA genes with highest variance in expression across the seven neuronal populations. Cell types are clustered based on Euclidean distance. 18/25 of the high variance tRNA genes are also significantly differentially expressed between group 1 and group 2 neurons (q value ≤0.05 and $|Log_2FoldChange| > 0.5$). High variance tRNA genes that are not differentially expressed between group 1 and group 2 neurons are labeled in bold.

(B) Composition of tRNA isoacceptor families with high-variance members. Note the high expression of *tRNA-Arg-TCG-3-1* and *tRNA-Pro-CGG-1-1*, and the low expression of *tRNA-Ala-TGC-4-1* in cerebellar granule cells relative to other neuronal populations. In addition, note the relatively higher level of *tRNA-Ser-GCT-1-1* in Purkinje cells and the low expression of *tRNA-Ala-TGC-4-1* in microglia relative to the other non-neuronal cell populations in the nervous system. The relative contributions of individual members of tRNA families to the global tRNA pool (mean + SEM) are shown in the indicated colors. The liver is included as a non-nervous system outgroup. N1: group 1 neurons, N2: group 2 neurons, NN: non-neuronal cells.

(C) Heatmap of the Euclidean distance between expression of tRNA genes in the seven analyzed neuronal populations.

(D) Heatmap of the *Z* scores for expression of the 15 tRNA genes with highest variance in expression across the four group 1 neuronal populations (Purkinje cells, dopaminergic neurons, spinal cord cholinergic neurons and layer V cortical neurons). Cell types are clustered based on Euclidean distance.

(E) Heatmap of the *Z* scores for expression of the 25 tRNA genes with highest variance in expression across the four non-neuronal cell types in the nervous system (astrocytes, oligodendrocytes, microglia and pericytes). Cell types are clustered based on Euclidean distance.

Supplementary Figure S4

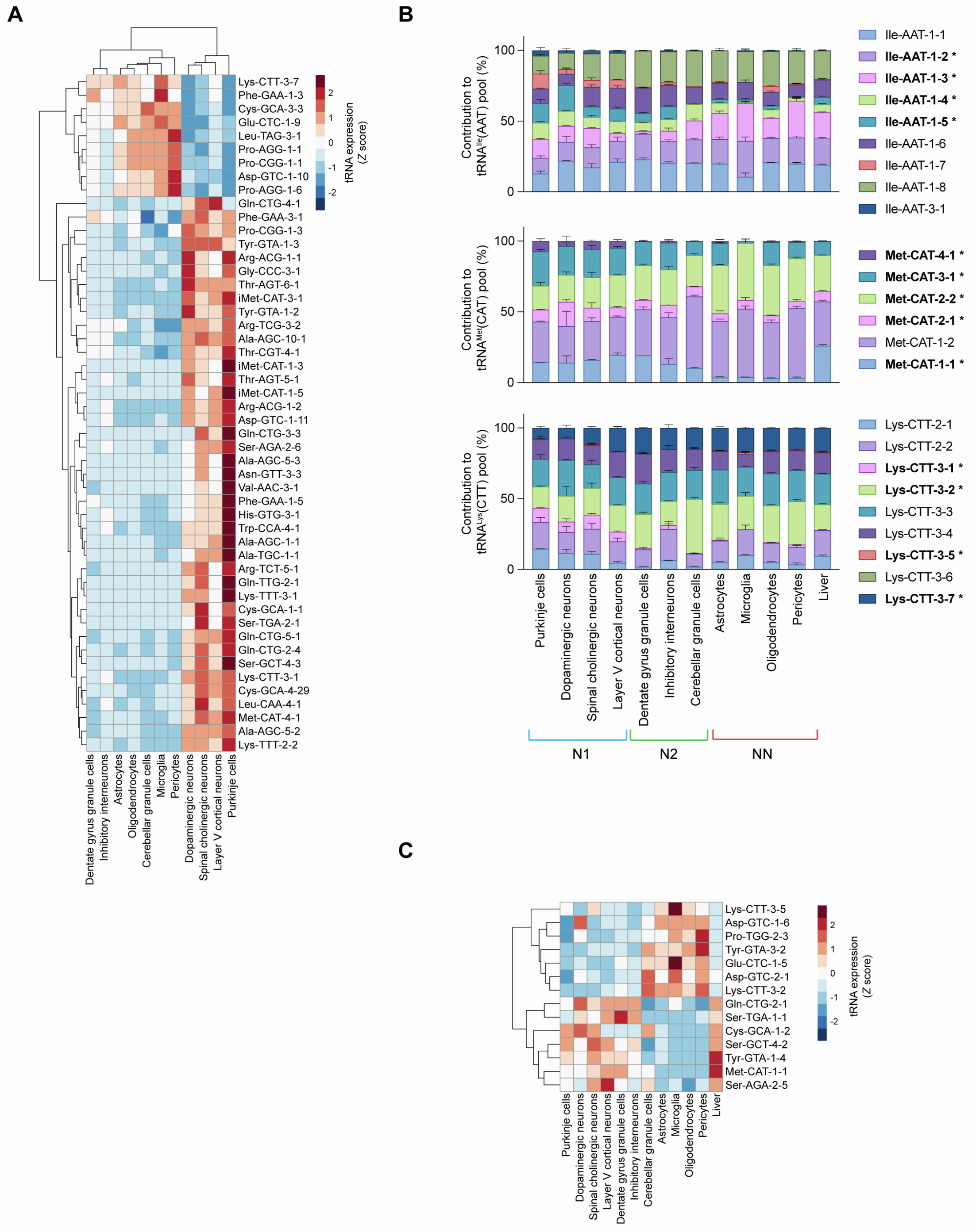

Figure S4. Differential expression of tRNA genes between neuronal and non-neuronal cells in the nervous system, related to Figure 3.

(A) Heatmap of *Z* scores for the expression of tRNA genes that are specifically enriched in group 1 neurons relative to non-neuronal cells. tRNA genes that are differentially expressed (q value ≤0.05 and $|Log_2FoldChange| > 0.5$) between neuronal and non-neuronal cells, and between group 1 and group 2 neurons are shown. Columns are clustered based on Euclidean distance.

(B) Composition of tRNA isoacceptor families containing both neuronal and non-neuronal enriched isodecoders. tRNA genes that are differentially expressed between neuronal and non-neuronal cell types are bolded and labeled with an asterisk. Ile-AAT: *tRNA-Ile-AAT-1-4* and *1-5* are upregulated in neurons, while *tRNA-Ile-AAT-1-2* and *1-3* have higher expression in non-neuronal cells. Met-CAT: *tRNA-Met-CAT-1-1*, *2-1*, *3-1* and *4-1* are upregulated in neurons, while *tRNA-Met-CAT-2-2* is upregulated in non-neuronal cells. Lys-CTT: *tRNA-Lys-CTT-3-1* is upregulated in neurons, while *tRNA-Lys-CTT-3-2* and *3-7* are upregulated in non-neuronal cells. The relative contributions of individual tRNA genes to their isoacceptor family (mean + SEM) are shown in the indicated colors. N1: group 1 neurons, N2: group 2 neurons, NN: non-neuronal cells.

(C) Heatmap of *Z* scores for the expression of tRNA genes whose expression differs between non-neuronal cells in the nervous system and liver.

Supplementary Figure S5

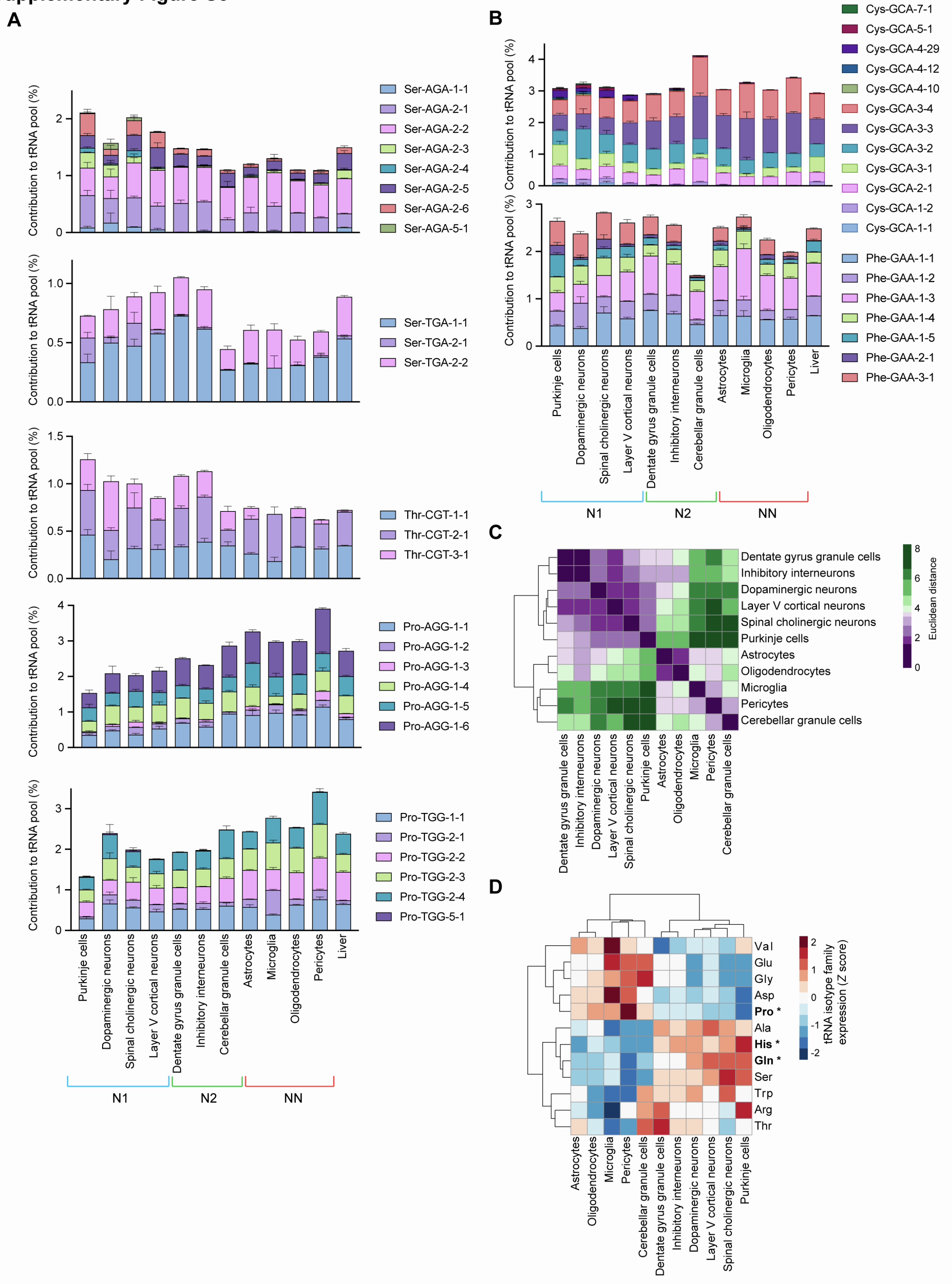

Figure S5. tRNA isoacceptor and isotype families are differentially expressed between neuronal and non-neuronal cells, related to Figure 4

(A) Composition of tRNA isoacceptor families that are enriched in neurons relative to non-neuronal cells (Ser-AGA, Ser-TGA, and Thr-CGT) and vice versa (Pro-AGG and Pro-TGG). The relative contributions of tRNA genes to the global tRNA pool are shown on the y-axis (mean + SEM). Individual members of the family are shown in the indicated colors. N1: group 1 neurons, N2: group 2 neurons, NN: non-neuronal cells.

(B) The composition of tRNA isoacceptor families whose levels in cerebellar granule cells deviate from other neuronal populations and non-neuronal cells. The relative contributions of tRNA genes to the global tRNA pool are shown on the y-axis (mean + SEM). Individual members of the family are shown in the indicated colors. N1: group 1 neurons, N2: group 2 neurons, NN: non-neuronal cells.

(C) Heatmap of the Euclidean distance between the expression of tRNA isotype families across the 11 analyzed cell types in the nervous system.

(D) Heatmap of the *Z* scores for expression of the 12 tRNA isotype families that are differentially expressed between neuronal and non-neuronal cells (q value ≤ 0.05). tRNA isotype families that met our minimum fold change threshold ($|Log_2FoldChange| > 0.5$) are highlighted in bold and labeled with an asterisk. Note that cerebellar granule

cells cluster with non-neuronal cells for the majority of these tRNA isotypes, except tRNA-Trp, tRNA-Arg, and tRNA-Thr.

Supplementary Figure S6

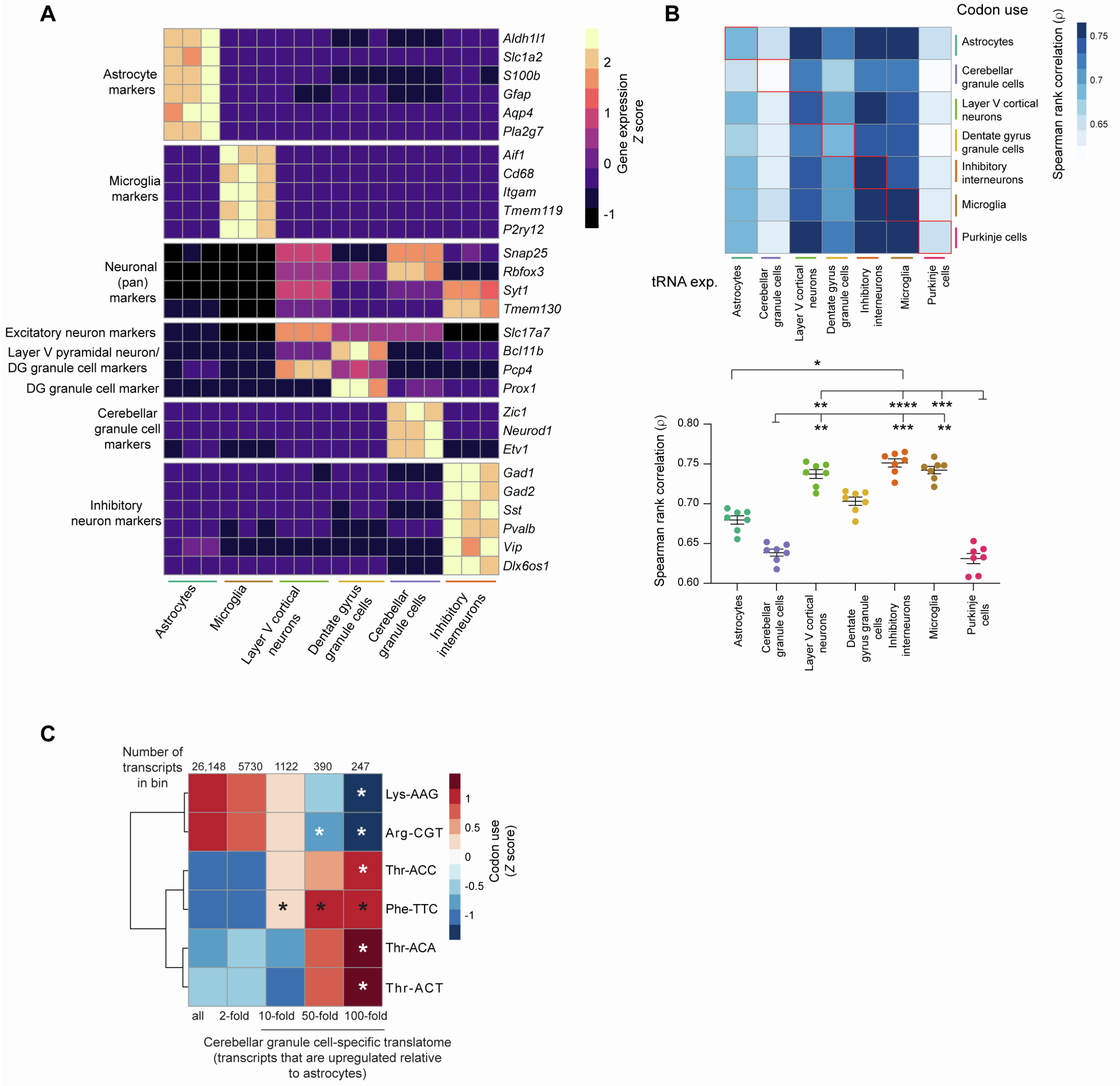

Figure S6. Analysis of the translatome in nervous system cell populations, related to Figure 5

(A) Heatmap of the *Z* scores of expression of well-established marker genes in the translatome of 6 nervous system cell types (gene expression in the appropriate RiboTag immunoprecipitated mRNA). Each cell type has three biological replicates.

(B) Above: Heatmap of Spearman rank correlation coefficient values between codon usage in the translatome and tRNA isoacceptor family levels (determined by ChIP-Seq) of the indicated nervous-system cell types. The codon usage values are the average of three biological replicates. The diagonal (outlined in red) from top left to bottom right shows the correlation between matched (i.e., the same cell type) codon use and tRNA expression. Below: Spearman rank correlation values between tRNA isoacceptor family levels in the indicated cell type and codon usage in all 7 nervous system cell types. Kruskal-Wallis with Dunn's post-test. * $p \leq 0.05$ ** $p \leq 0.01$ *** $p \leq 0.001$ **** $p \leq 0.0001$

(C) Heatmap of *Z* scores for codon usage in transcripts that are highly enriched in cerebellar granule cells relative to astrocytes. Permutation tests were used to determine whether codon usage in any given bin deviated significantly from that of an equivalent number of genes randomly selected from the total translatome (sampled 10,000 times). Empirical p values were calculated as the fraction of random gene sets of the same size whose codon usage was either higher or lower than that of the bin of interest. Bins in which the codon usage was significantly different than expected based on random

sampling of the transcriptome are labeled with an asterisk (*). Codon usage in the indicated total cellular translatome (all) or in subsets of transcripts that are upregulated relative to the second cell type (2-fold, 10-fold, 50-fold,100-fold) is shown. The number of transcripts in each bin is indicated.

Supplementary Figure S7

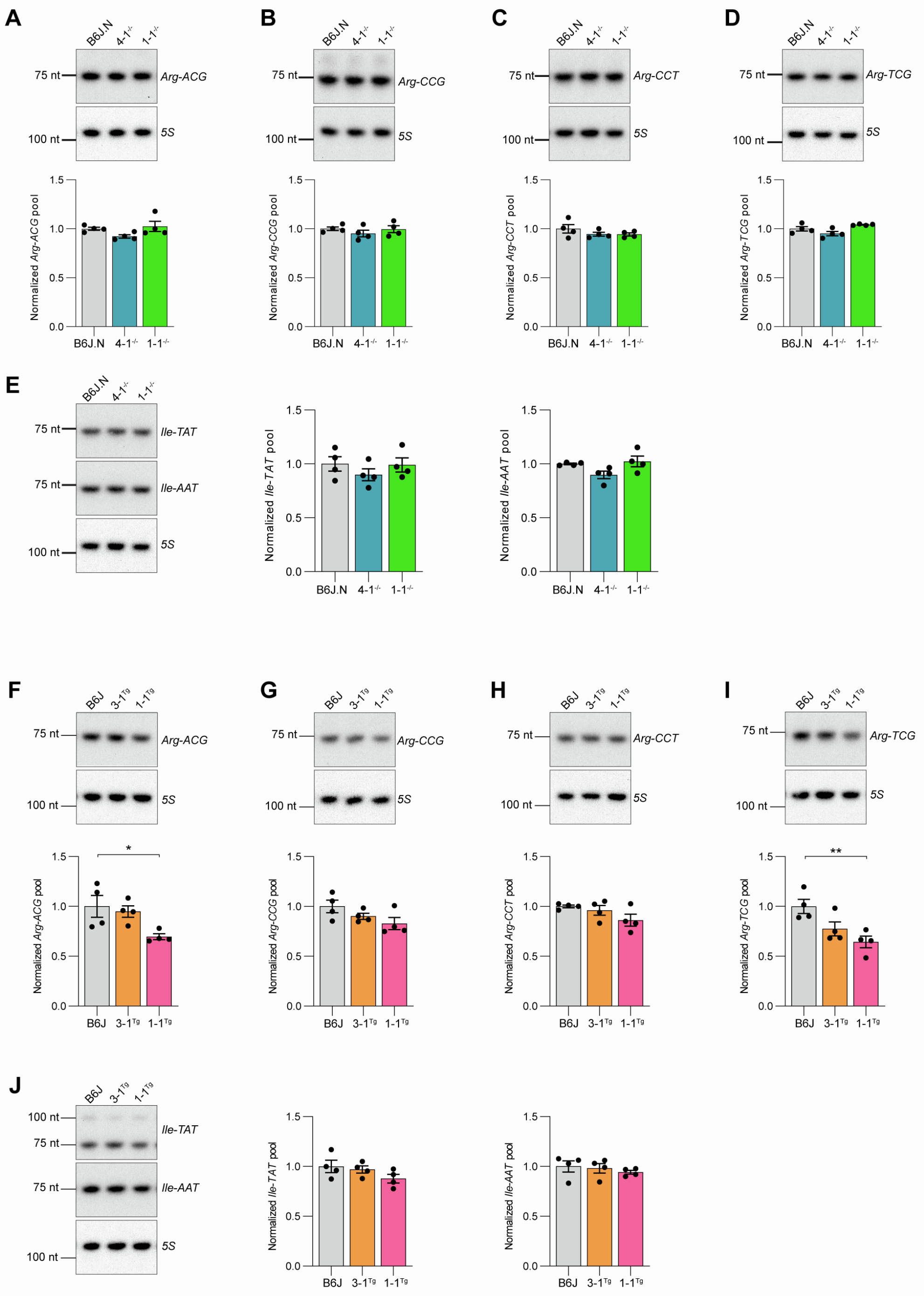

Figure S7. Overexpression of *tRNA-Arg-TCT-3-1* and *tRNA-Arg-TCT-1-1* results in a significant decrease in the levels of other arginine tRNA isoacceptor families, related to Figure 6

(A-E) Northern blots examining the expression of the remaining $tRNA^{Arg}$ isoacceptor families and all $tRNA^{Ile}$ isoacceptor families following mutagenesis of *tRNA-Arg-TCT-4-1* and *tRNA-Arg-TCT-1-1* in the cerebellum. The expression was compared to congenic B6J mice in which the wild-*type tRNA-Arg-TCT-4-1* gene was transferred from B6N (B6J.N).

(F-J) Northern blots examining the expression of the remaining $tRNA^{Arg}$ isoacceptor families and all $tRNA^{Ile}$ isoacceptor families in the cortex of transgenic mice overexpressing *tRNA-Arg-TCT-3-1* or *tRNA-Arg-TCT-1-1* compared to C57BL/6J (B6J) controls.

(A-J) Expression was assessed using pooled probes overlapping gene-specific SNPs or a probe common to all tRNA genes in the respective family. Bands were normalized to 5S rRNA and quantified relative to respective controls. Data are reported as mean ± SEM. One-way ANOVA with Tukey post-test * $p \leq 0.05$, ** $p \leq 0.01$, *** $p \leq 0.001$, **** $p \leq 0.0001$

Supplementary Figure S8

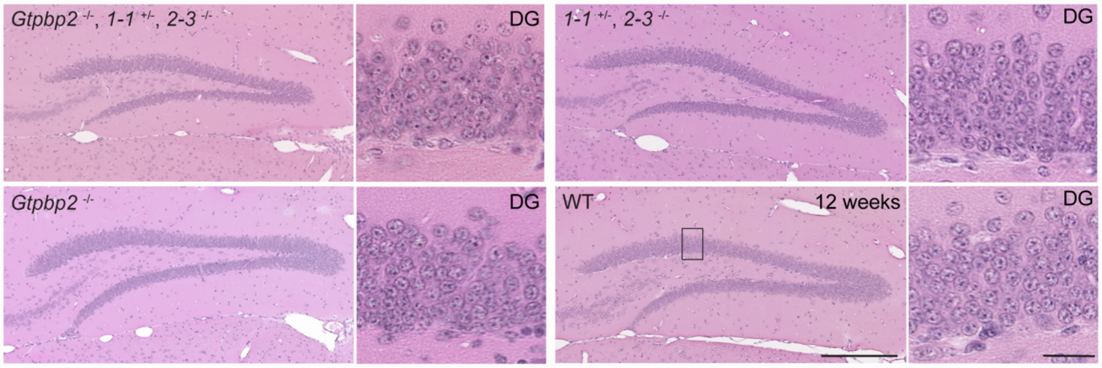

Figure S8. Depletion of the tRNA^{Ile}(TAT) pool in *Gtpbp2*$^{-/-}$ mice does not cause degeneration of dentate gyrus granule cells, related to Figure 7

Hematoxylin– and eosin–stained (H&E) sagittal sections of the dentate gyrus from 12-week-old mice. Higher magnification images of the region indicated by the black rectangle in WT are shown for each genotype. Scale bar: 250 µm and 25 µm (higher magnification).
